# Hyperpolarization Modulation of the T‐Type hCa_v_3.2 Channel by Human Synenkephalin [1–53], a Shrew Neurotoxin Analogue without Paralytic Effects

**DOI:** 10.1002/anie.202503891

**Published:** 2025-05-02

**Authors:** Ryo Fukuoka, Yusuke Yano, Nozomi Hara, Chihiro Sadamoto, Andres D. Maturana, Masaki Kita

**Affiliations:** ^1^ Department of Applied Biosciences, Graduate School of Bioagricultural Sciences Nagoya University Furo‐cho, Chikusa Nagoya 464–8601 Japan; ^2^ Promotion Office for Open Innovation, Institutes of Innovation for Future Society Nagoya University Furo‐cho, Chikusa Nagoya 464–8601 Japan

**Keywords:** Molecular dynamics simulation, Natural venom analogues, Peptide synthesis, Synenkephalin, T‐type Ca channel modulators

## Abstract

Mammalian secreted venoms mainly consist of peptides and proteases used for defense or predation. *Blarina* paralytic peptides (BPPs), mealworm‐targeting neurotoxins from shrew, are very similar to human synenkephalin. This peptide is released from proenkephalin in the brain along with opioid peptides that mediate analgesic and antidepressant effects, though its physiological function is unclear. Here, we synthesized and characterized human synenkephalin [1–53] (hSYN) and reveal its disulfide bond connectivity. Similar to BPP2, hSYN caused a hyperpolarizing shift in the human T‐type voltage‐gated calcium channel (hCa_v_3.2) at 0.74 µM, but did not paralyze mealworms. Molecular docking and molecular dynamics simulations showed that hSYN and BPP2 interact with hCa_v_3.2 channel differently, due to differences in polar residues. Since Ca_v_3.2 channel regulates neuronal excitability and is implicated in conditions like autism and epilepsy, our findings on hSYN could provide insight into the channel gating and agonistic mechanisms, along with potential pathways for developing treatments for neurological disorders.

## Graphical abstract

Human synenkephalin [1–53] (hSYN), an analogue peptide of shrew saliva neurotoxins, was synthesized and its structural characteristics studied. Synthetic hSYN potently activated the T‐type voltage‐gated Ca channel hCa_v_3.2 but did not paralyze mealworms. These findings offer new insight into neurological disorder treatment and evolutionary mechanisms of venom peptides in mammals.

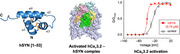

## Introduction

Bioactive peptides in mammals, including humans, play roles in various physiological and behavioral activities, such as feeding and body weight regulation, sleep wake cycles, reproduction, anxiety, depression, pain, and maintaining homeostasis.^[^ ^1^ ^]^ Both forward and reverse genetics approaches have revealed the relationships between diseases and receptor mutations in the signal transduction of bioactive peptides. Furthermore, based on the properties of endogenous ligands and natural toxins that specifically interact with these receptors, many agonistic and antagonistic peptides have been developed and applied clinically.^[^ ^2^, ^3^, ^4^ ^]^ For example, ω‐conotoxin, a paralytic toxin from cone snails, acts on ion channels that are important for neurotransmission and pain perception. Elucidation of the inhibitory mechanism of the voltage‐gated N‐type calcium channel (Ca_v_2.2) led to the development of the analgesic drug Ziconotide.^[^ ^5^, ^6^, ^7^, ^8^, ^9^, ^10^ ^]^ A variety of peptides from scorpion venom have also contributed to the development of medicines with analgesic, antiepileptic, and immunoregulation properties.^[^ ^11^, ^12^ ^]^

Recent advances in genomics have led to the acquisition of enormous amounts of data on gene functions, providing a comprehensive understanding of biomolecules. However, predicting the functions of endogenous and exogenous peptides with low similarity to known ligands and identifying their target molecules remain major challenges. During evolution, venomous animals developed specialized substances that provide survival advantages involving defense mechanisms and predation.^[^ ^13^, ^14^ ^]^ Although fewer in number than the number of toxin‐producing animals such as insects and reptiles, certain mammals such as shrews and platypus produce their own secretory toxins rather than simply accumulating them from external sources.^[^ ^15^, ^16^, ^17^ ^]^ Studies including from our group have shown that the main components of mammalian venoms are peptides or proteases.^[^ ^18^, ^19^, ^20^, ^21^, ^22^ ^]^ These molecules often arise from structural mutations in hormones and enzymes commonly found in mammals, and serve as chemical weapons against their enemies, competitors, and prey.^[^ ^23^, ^24^ ^]^ From this perspective, focusing on the unique structures and functions of exogenous peptide toxins may provide a starting point for exploring the unknown functions of similar endogenous peptides, which may bring new insights into animal and human biology.

**Figure 1:**
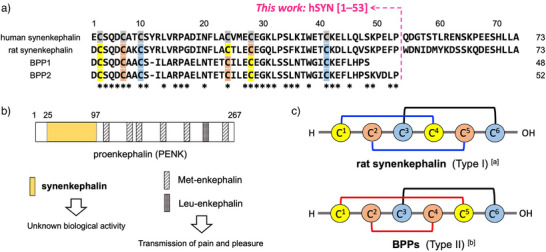
Structures of synenkephalins (proenkephalin (PENK) [25–97]) and *Blarina* paralytic peptides (BPPs). a) Sequence alignment of human and rat synenkephalins, and *Blarina* paralytic peptides (BPPs 1 and 2). Asterisks indicate conserved residues among synenkephalins and BPP2. b) Position of synenkephalin and opioid peptides in PENK. c) The two types of disulfide bond connectivity (Types I and II). [a] See Ref. [50]. [b] See Ref. [28].

The short‐tailed shrew *Blarina brevicauda* is one of the rare venomous mammals that has potent neurotoxins in its saliva and effectively captures prey such as earthworms and small insects.^[^ ^25^ ^]^ Since *Blarina* shrew venom causes severe pain in those bitten,^[^ ^26^ ^]^ it was predicted that the venom targets specific ion channels related to pain and neurotransmission.^[^ ^27^ ^]^ Recently, we isolated and characterized *Blarina* paralytic peptides (BPPs) 1 and 2 as major active substances against mealworms (Figure 1a).^[^ ^28^ ^]^ BPP2 was chemically synthesized based on the gene sequence of the precursor protein proenkephalin (PENK).^[^ ^29^ ^]^ Synthetic BPP2 was identical to the natural compound but had no significant effect on hCa_v_2.2, and instead showed a hyperpolarization shift (–11 mV) in the activation of human T‐type calcium channel (hCa_v_3.2) at 0.84 µM.

T‐type calcium channels (Ca_v_3.1, 3.2, 3.3) are low‐threshold voltage‐gated channels with fast inactivation and slow deactivation rates.^[^ ^30^ ^]^ All three subtypes are widely expressed in the nervous, neuroendocrine, and cardiovascular systems, and play important roles in sleep homeostasis, the response to pain, and the development of epilepsy.^[^ ^31^, ^32^, ^33^ ^]^ Among them, Ca_v_3.2 is widely expressed in the central nervous system, where it controls neuronal excitability and is involved in pain sensation. This channel also plays an important role in repetitive firing in the brain and heart, and its mutations are directly linked to several pathologies, including autism, amyotrophic lateral sclerosis, Alzheimer's disease, and epilepsy.^[^ ^34^, ^35^, ^36^ ^]^ Therefore, the discovery of Ca_v_3.2‐specific modulators and understanding their gating and voltage regulation mechanisms may be beneficial for the development of therapeutic drugs for various neurological disorders.

BPPs 1 and 2 have a single peptide chain of 47 and 52 amino acids, respectively, with a common N‐terminal sequence. BPPs have three intramolecular disulfide bonds via six Cys residues. The amino acid sequences of BPPs show high similarity to a portion of synenkephalin that is ubiquitously expressed in the brain and adrenal gland, corresponding to the N‐terminal 25–97 residues of PENK (Figure 1b).^[^ ^37^, ^38^ ^]^ When PENK is processed, non‐opioid synenkephalin is released along with methionine‐enkephalin (Met‐enkephalin) and leucine‐enkephalin (Leu‐enkephalin).^[^ ^39^ ^]^ Both Met‐enkephalin and Leu‐enkephalin bind to opioid receptors expressed in the brain and spinal cord, and mediate analgesic and antidepressant effects.^[^ ^40^ ^]^ Synenkephalin has also been detected in abundance in the rat embryonic brain, and is thought to have some function in developing neurons,^[^ ^41^ ^]^ but its physiological role remains unclear.^[^ ^42^, ^43^, ^44^ ^]^ Processing of PENK and the release of fragmented synenkephalin has also been observed in human and rat bone marrow and splenic mononuclear cells.^[^ ^45^, ^46^ ^]^ However, synenkephalin has not been found in the saliva or salivary glands of most mammals, except for several shrews^[^ ^29^, ^47^, ^48^ ^]^ and solenodons,^[^ ^49^ ^]^ suggesting that BPPs and related peptides are specifically secreted in saliva by insectivorous mammals.

The disulfide bond connectivity of rat synenkephalin has been reported to be Cys(I)–Cys(IV), Cys(II)–Cys(V), and Cys(III)–Cys(VI) (termed Type I) based on an analysis of the recombinant protein by enzymatic digestion (Figure 1c).^[^ ^50^ ^]^ The disulfide bond pattern of human synenkephalin has not been experimentally confirmed, but its Type I structure has been deposited in several databases due to its high homology (74%) with rat synenkephalin and their conserved Cys residues. Meanwhile, we established that BPP2 has alternative disulfide bond connectivity, Cys(I)–Cys(V), Cys(II)–Cys(IV), and Cys(III)–Cys(VI) (termed Type II), by comparison of the data with those of natural and synthetic compounds.^[^ ^28^ ^]^ These different disulfide bond patterns were expected to arise because BPPs have 21–26 fewer residues at the C‐terminus compared to synenkephalins, or because the residue differences result in different stable three‐dimensional structures. Alternatively, the surrounding Cys residues could induce different folding patterns during the formation of mature synenkephalin, but the reasons for these differences are currently unknown.

Based on the above background, we aimed to synthesize human synenkephalin N‐terminal part [1–53] (termed hSYN) as a human analogue of BPPs, and explore its stable structure, function and significance. We also aimed to verify the relationship between the activation of hCa_v_3.2 channel and the paralytic effect on mealworms by functional evaluation of hSYN. In addition, based on the cryo‐electron microscopy (Cryo‐EM) structure of the hCa_v_3.2 channel,^[^ ^34^ ^]^ we simulated the binding modes of hSYN and BPP2 on this channel. By clarifying their similarities and differences, our study sought to understand the chemical evolution and diversity of mammal‐specific exogenous neurotoxins and endogenous substances.

## Results and Discussion

### Synthesis of hSYN and Disulfide Bond Connectivity Analysis

**Scheme 1:**
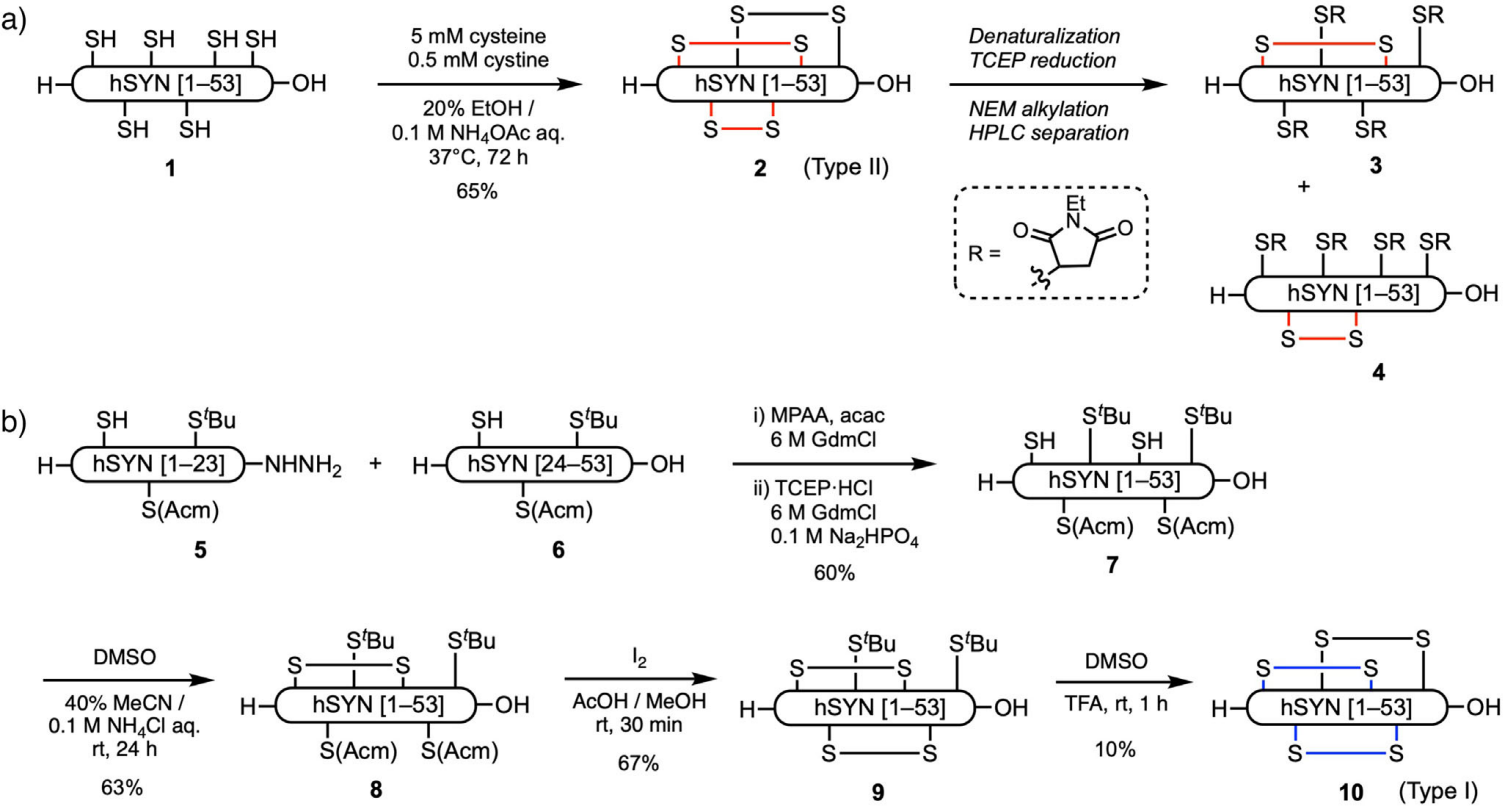
Synthesis of human synenkephalin analogue [1–53] (hSYN). a) Preparation of Type II hSYN (**2**) and its partial degradation for the analysis of disulfide bond connectivity. b) Preparation of Type I hSYN (**10**) by stepwise disulfide bond formation. Acm, acetamidomethyl; Gdm·HCl, guanidine hydrochloride; MPAA, 4‐mercaptophenylacetic acid; NEM, *N*‐ethylmaleimide; TCEP, tris(2‐carboxyethyl)phosphine.

We first examined the preparation of hSYN from the linear precursor peptide **1** (Scheme 1a). Oxidation of **1** in air or with DMSO gave only misfolded analogues with two (2SS) and/or three (3SS) disulfide bonds (Figure S1a,b). Meanwhile, a redox condition using cysteine / cystine in EtOH / aq. NH_4_OAc^[^ ^51^ ^]^ smoothly promoted the formation of three disulfide bonds to afford hSYN (**2**) in 65% yield almost as a single isomer, along with trace amounts of misfolded 3SS analogue. These misfolded products were predominantly converted to **2** under the same conditions as mentioned above, which was found to be the most thermodynamically stable (Figure S1c,d).

To determine the connectivity of disulfide bonds in synthetic **2**, enzymatic digestion and subsequent fragment peptide analysis were performed. The core N‐terminal 1–41 part of BPP2, which contains three disulfide bonds, is highly stable toward trypsin or glutamyl endopeptidase (Glu‐C) digestion.^[^ ^28^ ^]^ On the other hand, intact **2** was partially digested under the same conditions due to the presence of several reactive site residues (Arg12, Lys36, and Glu39), which is different from BPP2 (Figures S2 and S3). However, due to high proteolytic stability, analysis of these degraded products provided little information on the disulfide bond connectivity in **2**. To perform stepwise *S*‐alkylation with limiting reduction of disulfide bonds,^[^ ^52^, ^53^ ^]^ **2** was denatured with 6 M aq. guanidine hydrochloride (GdmCl)/0.1 M aq. citrate, followed by tris(2‐carboxyethyl)phosphine (TCEP) reduction and Michael addition to *N*‐ethylmaleimide (NEM). As a result, we obtained six kinds of *S*‐alkylated products (2SS‐a/b/c and 1SS‐a/b/c) with one or two disulfide bonds cleaved, respectively, along with unreacted **2** (3SS) and a fully *S*‐alkylated product (0SS) (Scheme 1a and Figure S4a). HPLC‐purified 1SS‐a (**3**) and 1SS‐b (**4**), which have different patterns of four Cys(*S*‐Nem) residues, were then reduced with DTT and alkylated with iodoacetamide. MALDI MS/MS analysis of the tryptic peptide fragments revealed that Cys2/Cys28 and Cys6/Cys24 were the paired residues to form disulfide bonds in **3** and **4**, respectively (Figures S4b, S5, and S6). Thus, the disulfide bond connectivity of synthetic **2** was shown to be Type II, identical to that of BPP2.

For comparison, Type‐I hSYN (**10**) was also synthesized by stepwise disulfide bond formation (Scheme 1b). The N‐terminal [1–23] hydrazine segment **5** and the C‐terminal [24–53] cysteine segment **6**, in which the Cys residues are protected with Acm and *^t^* Bu groups, were prepared by conventional Fmoc solid‐phase peptide synthesis. Native chemical ligation^[^ ^54^ ^]^ of **5** and **6** afforded the linear peptide **7** in 60% yield, using 4‐mercaptophenylacetic acid (MPAA) and acetylacetone for thioesterification and TCEP⋅HCl as the reducing agent.^[^ ^55^ ^]^ Oxidation of **7** with aqueous DMSO with NH_4_Cl and MeCN afforded the 1SS product **8** with the Cys(I)–Cys(IV) connectivity in 63% yield. Oxidative removal of the Acm groups and subsequent disulfide bond formation with iodine in AcOH/MeOH^[^ ^56^ ^]^ gave the 2SS product **9** with the Cys(II)–Cys(V) connectivity in 67% yield. Finally, removal of the *^t^* Bu group and simultaneous disulfide bond formation by DMSO/TFA^[^ ^57^ ^]^ afforded **10** with the Cys(III)–Cys(VI) connectivity in 10% yield, despite the problems with the oxidation of Met26 and Trp38 residues and subsequent degradation of the polypeptide chain. In capillary C_18_ reversed‐phase (RP)‐HPLC analysis, **10** eluted earlier than **2** (*t*_R_ 17.6 and 18.8 min, respectively) (Figure S7a), which indicated that **10** was different than the major 3SS analogues obtained from **1** by air or DMSO oxidation under kinetic control (Figure S1b).

We cannot completely exclude the possibility that disulfide reshuffling occurred during partial reduction with TECP in **2**. Thus, the Glu‐C digestion products of **2** and **10** without disulfide bond reduction were analyzed by LC‐MS (Figure S7b,c). The retention times of the 3SS products [1–29, 40–43] between **2** and **10** were quite different, which showed that original **2** and **10** had different disulfide bond patterns. These results suggested that our proposed disulfide bond connectivity of **2** was reasonable.

### Biological Activity of hSYN

**Figure 2:**
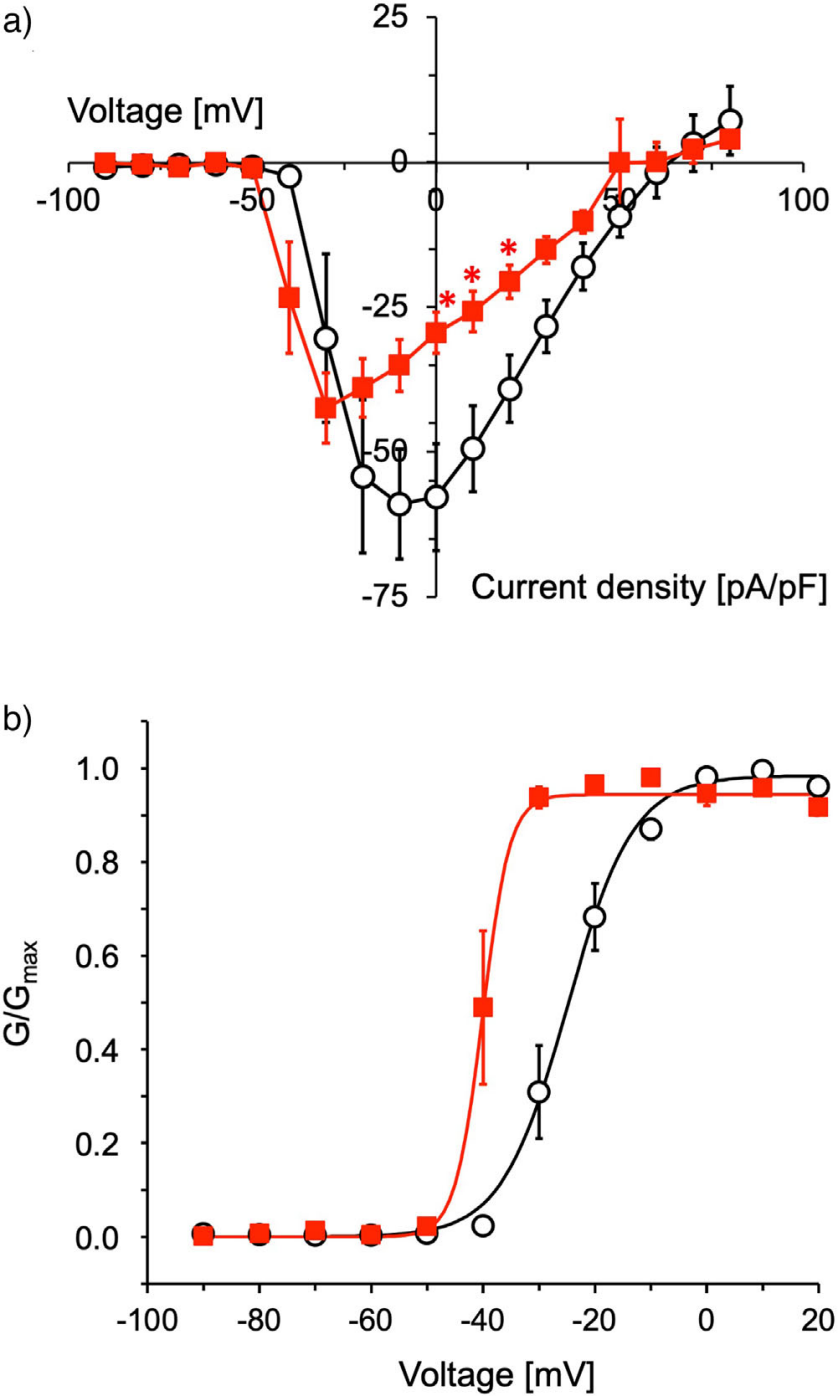
Voltage‐gated Ca^2+^ currents measured by whole‐cell patch clamp experiments using HEK293T cells expressing hCa_v_3.2. a) The current density–voltage relationships were recorded before (○) or after (■) addition of synthetic Type‐II hSYN (**2**) (0.74 µM, *n* = 5). b) Steady‐state activation curve of hCa_v_3.2. Normalized conductance (*G*/*G*_max_) was generated for both data sets, fit to a Boltzmann equation, and is shown as data ± S.E.M. (*n* = 5). Statistical analyses were performed using Dunnett's multiple comparison tests. ^*^ *p* < 0.05 versus control.

hCa_v_3.2 is known to initiate signals at peripheral nerve endings in nociceptive pathways.^[^ ^58^ ^]^ To better understand the sequence‐activity relationship with BPP2, we examined the effect of a human analogue on this channel. Notably, synthetic hSYN (**2**) showed a hyperpolarization shift (−15 mV) of hCa_v_3.2 activation at 0.74 µM (Figure 2). In whole‐cell patch‐clamp experiments, the lowest current density peak (ca. −42 pA/pF) was observed at −30 mV, which is a lower voltage than those in control (ca. −59 pA/pF at −10 mV). These results showed that **2** enhances the sensitivity of hCa_v_3.2 channel to a change in membrane potential, similar to the actions of several gating‐modifier peptide toxins,^[^ ^59^ ^]^ such as huwentoxin‐IV^[^ ^60^ ^]^ and vanillotoxins,^[^ ^61^, ^62^, ^63^ ^]^ on other channels. In addition, at 0 to +20 mV, current density was significantly reduced by **2**, suggesting a block at higher membrane depolarization.

Synthetic BPP2 reliably induces paralysis and convulsions in mealworms (larva of *Zophobas atratus*) immediately after intraperitoneal administration (5.6 µg g^−1^ body weight).^[^ ^28^ ^]^ In contrast, synthetic hSYN (**2**) had no paralytic effects on mealworms, even after administration of a much higher amount (50 µg g^−1^ body weight) than BPP2 (Table 1). These results suggested that the T‐type Ca channel activation of **2** and BPP2 are not directly related to the immediate paralytic effect on mealworms caused by BPP2. Although intact hSYN (**2**) was susceptible to several enzymes, its degradation was much slower than the paralytic effect of BPP2 on mealworms.^[^ ^28^ ^]^ Thus, differences in the proteolytic stability between hSYN and BPP2 are expected to have little effect on paralytic activity.

**Table 1:** Paralytic activity of synthetic hSYN (**2**) and BPP2 against *Zophobas atratus* (larva) by intraperitoneal (*i.p*.) injection.

Sample	Dose (µg/g bodyweight)	Paralyzed/total numbers
Control^a)^	–	0/3
**2**	5.0	0/4
**2**	15	0/3
**2**	50	0/4
BPP2	5.6	3/3^b)^

^a)^ PBS (100 µL per 1 g mealworm bodyweight) was injected.

^b)^ See Ref. [28].

### Comparison of the Three‐Dimensional Structures of hSYN and BPP2

**Figure 3:**
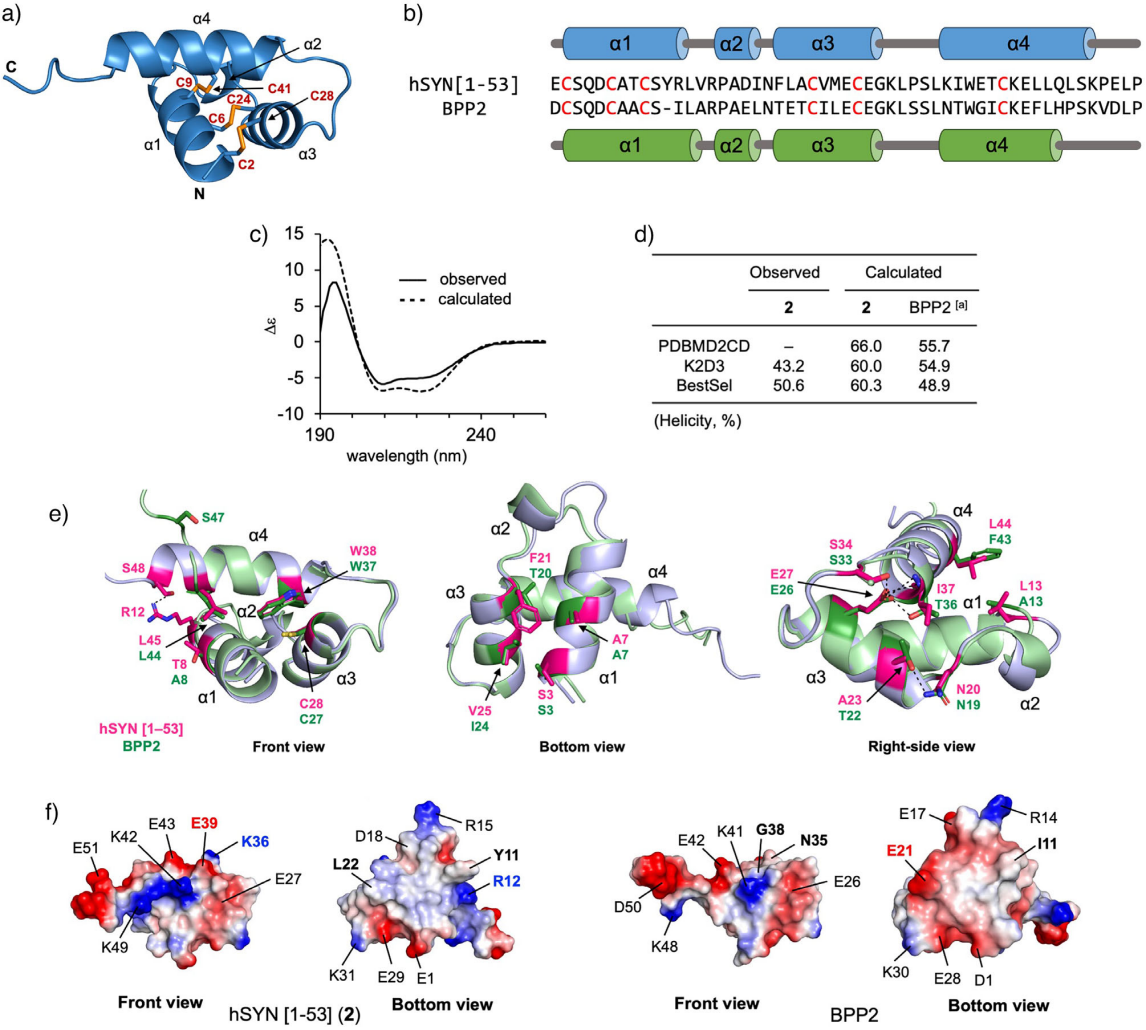
Proposed three‐dimensional structures of hSYN and BPP2. a) ColabFold‐predicted structure of hSYN. Disulfide bonds are highlighted in orange sticks. b) Proposed helices in the calculated structures. c) CD spectra of hSYN. Obs: Synthetic **2** (20 µM) was measured in 50 mM potassium phosphate (pH 7.0) at 25 °C. Calc: The ColabFold‐predicted structure was analyzed using the PDBMD2CD server. d) Calculated helicities (%) of each model by three methods based on the CD spectra. e) Superimposed structures of hSYN (blue white) and BPP2 (pale green). Key residues that interact between the helixes are shown in stick models. Polar interactions are shown by black dotted lines. f) Surface charge models shown by a red (negative) – blue (positive) heat map analysis. Bold residues indicate a characteristic difference in hSYN and BPP2. [a] See Ref. [28].

To compare the structural properties of hSYN and BPP2, we next examined their three‐dimensional structures using ColabFold^[^ ^64^ ^]^ (a Google Colab version of AlphaFold2^[^ ^65^ ^]^ using MMSeq2^[^ ^66^ ^]^). This analysis suggested that hSYN has a helix‐rich structure with the Type‐II disulfide bond connectivity identical to that in **2** and BPP2 (Figures 3a and S8A–C). The predicted N‐terminal core domain structure of human synenkephalin also showed high similarity to **2**, despite having a fluctuating random coil structure at the C‐terminus (Figure S8D–F). The four helical structures of hSYN were highly similar to those of BPP2, except for the extended C‐terminus (Gln46–Ser48) in the α4 helix (Figure 3b). In the CD spectrum of synthetic **2**, two negative bands at 210 and 219 nm and a positive band at 194 nm were observed, which are characteristic of α‐helix‐rich structures (Figure 3c).^[^ ^67^ ^]^ This observed CD spectrum was in good agreement with that calculated from the ColabFold‐predicted structure of **2** by the PDBMD2CD server,^[^ ^68^ ^]^ and its helicity (66.0%) was greater than that of BPP2 (55.7%) (Figure 3d). The calculated helicities of hSYN estimated by the K2D3^[^ ^69^ ^]^ and BestSel^[^ ^70^ ^]^ servers (60.0 and 60.3%, respectively) were also higher than those of BPP2 (54.9 and 48.9%).

The four helices of hSYN and BPP2 in ColabFold models are in almost the same orientation, due to the conserved hydrophobic interactions between the helices, such as Ser3/Val25, Ala7/Phe21, Thr8/Leu45, Leu13/Leu44, and Cys28/Trp38 as well as the electrostatic interactions with the polar residues Glu27/Ser34 and Glu27/Ile37 (main NH) for hSYN (Figure 3e). Meanwhile, due to the substitution of Ile11 on BPP2 by Tyr11–Arg12, hSYN had a unique polar interaction Arg12/Ser48, which may stabilize the extended α4 helix structure. As for the surface charge, Gln35 and Gly38 in BPP2 were replaced by polar residues Lys36 and Glu39 in hSYN (Figure 3f). In addition, the basic residue Arg12 in hSYN was missing in BPP2, and the acidic residue Glu21 in BPP2 was replaced by Leu22 in hSYN. As a result, the apparent pI value of hSYN (4.86) was increased compared to those of BPP1 (4.49) and BPP2 (4.55), which might cause the differences in the binding mode with the target receptor.

### Homology Modeling of the Human T‐Type Ca Channel hCa_v_3.2

**Figure 4:**
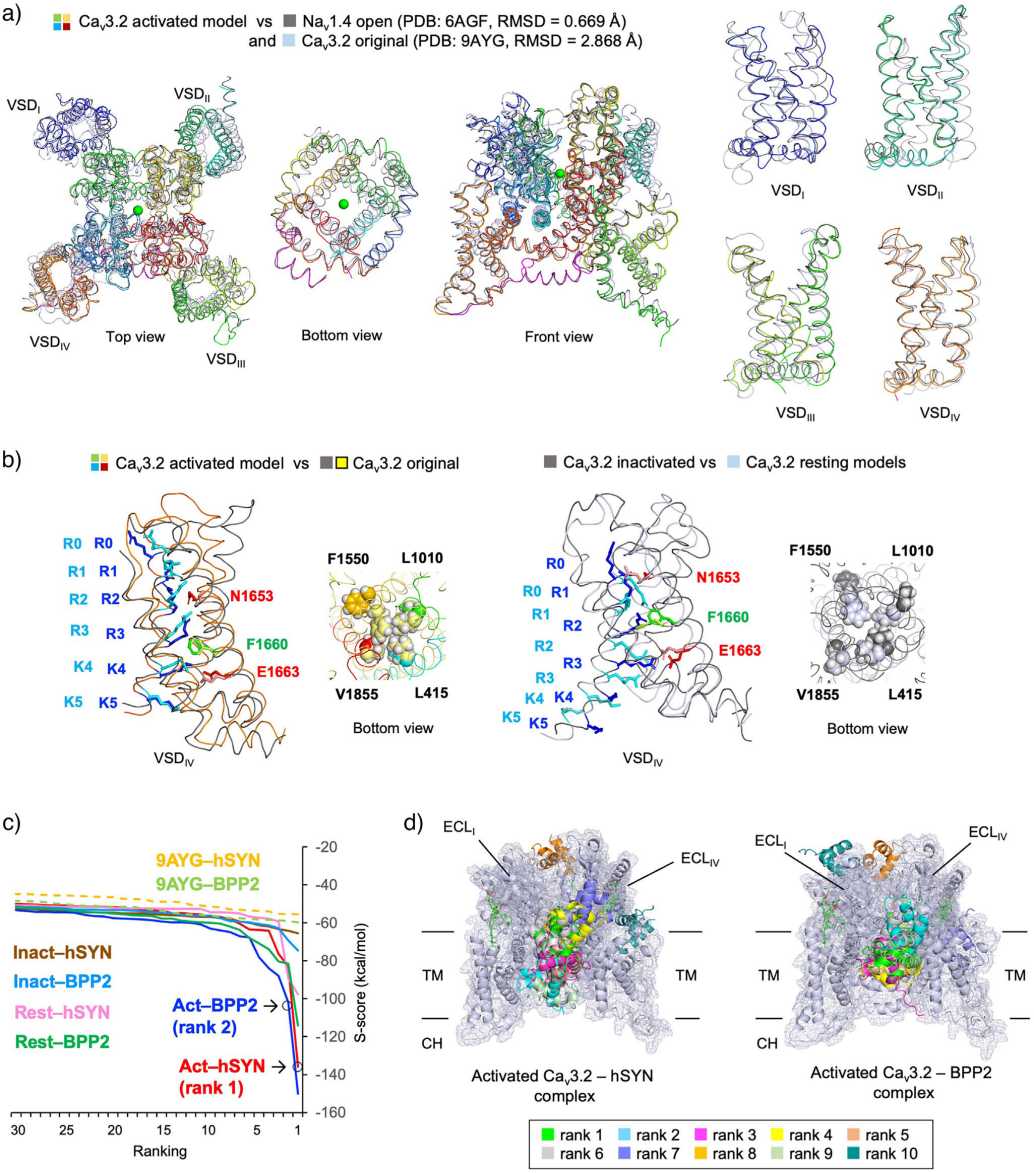
Proposed structures of hCa_v_3.2 channels and its complexes with hSYN and BPP2 obtained by molecular modeling studies. a) Structure of activated hCa_v_3.2 channel (multicolor) obtained by homology modeling with Na_v_1.4 opened channel (grey) as a template. The missing S3–S4 linker in the hCa_v_3.2 cyro‐EM structure is shown in magenta. b) Comparison of the VSD_IV_ and inner channel gate structures (cytosolic view) among the activated, inactivated, and resting hCa_v_3.2 channels obtained by homology modeling and the original hCa_v_3.2 channel (PDB: 9AYG). Characteristic gating positive‐charged residues at the S4 helix and their facing residues at the adjacent S2 and S3 helices are highlighted in stick models. c) Posing and scoring of the top 30 docking models based on the S‐scores. The activated hCa_v_3.2–hSYN (rank 1) and BPP2 (rank 2) complexes that were subjected to further MD simulations are highlighted in open circles. d) Superimposed structures of the top 10 PPI‐dock models of the activated hCa_v_3.2–hSYN and BPP2 complexes with the lowest docking scores (front view, see Figure S10 for details).

Voltage‐gated ion channels exhibit large structural changes among the resting, activated (open), and inactivated (close) states. Recently, cryo‐EM structures of human T‐type voltage‐gated calcium channels (hCa_v_3.1,^[^ ^71^ ^]^ 3.2,^[^ ^34^ ^]^ and 3.3^[^ ^72^ ^]^) were reported in apo or antagonist‐bound forms, but none of them captured the fully activated states. To establish the agonistic effects of hSYN and BPP2, we considered docking simulations with the three states of hCa_v_3.2 channel necessary. The core structures of Ca_v_ channels, including voltage‐sensing domains I∼IV [VSD_I∼IV_, with S1–S4 transmembrane (TM) helices] and pore domains I∼IV (PD_I∼IV_, with S5 and S6 helices), show high similarity to those of Na_v_ channels.^[^ ^73^ ^]^ The clockwise arrangement of the four homologous repeats from the extracellular view are conserved in all human Ca_v_ and Na_v_ channels. Therefore, using human Na_v_1.4 (activated state, PDB: 6AGF),^[^ ^74^ ^]^ American cockroach *Periplaneta americana* Na_v_1.4 (Na_v_PaS, inactivated state, PDB: 5X0M), and Campylobacteria *Arcobacter butzleri* Na_v_ (Na_v_Ab, resting state, PDB: 6P6W) channels as templates,^[^ ^75^ ^]^ three states of hCa_v_3.2 channel models with high similarity (RMSD = 0.669, 0.699, and 1.160 Å, respectively) were constructed by homology modeling using the Molecular Operating Environment (MOE) 2022.01 program package (Figures 4a, S9a,b). It is notable that in the activated hCa_v_3.2 channel model, the conformations of each TM helix on VSD_I∼IV_ and S1–S2 and S3–S4 extracellular loop (ECL) were highly different from the original ones.

To verify the structural differences among the three hCa_v_3.2 models, we focused on the positions of the gating positive‐charged residues R0–R5 (or K3, K4, K5) in the S4 helix, and their facing residues at the adjacent S2 and S3 helices that constitute the charge transfer center (Figures 4b and S9c). In the VSD_IV_ of an activated model, Phe1660 was located at R3/R4, and Asn1653 and Glu1663 had electrostatic interactions with R2 and K4, respectively. Thus, the VSD_IV_ exhibited an “up” conformation common to voltage‐gated ion channels in response to membrane depolarization. When the VSD_IV_ was superimposed, the above‐mentioned gating charged residues were in almost the same positions as in the original hCa_v_3.2 channel. In contrast, in the inactivated and resting models, Phe1660 was located at R1/R2, and R3, K4, and K5 were all below this occluding residue. Thus, the “down” conformation was generated by the large sliding motion of the S4 helix. These characteristic structural rearrangements of VSD_IV_, in which the gating charge residues are attracted to the cytoplasmic side, were also observed in other VSD_I∼III_ (Figure S9d). Regarding the inner channel gate (composed of Leu415, Leu1010, Phe1550, Val1855), the activated and inactivated models had open states, and the resting model had a half‐open state, whereas the original model had a closed state (Figure 4b). Furthermore, the S3–S4 linker, which is missing in the hCa_v_3.2 cryo‐EM structure, also showed a highly different orientation in the activated model compared with the inactivated and resting models (Figures 4a and S9a).^[^ ^75^ ^]^ These differences also support the validity of our proposed homology model structures.

### PPI‐Dock Models of the hCa_v_3.2–hSYN Complex

Based on the binding modes of related gating modifier toxins, we assumed that hSYN and BPP2 bind to the ECL domain of hCa_v_3.2 channel assembled in the plasma membrane. Therefore, we performed protein–protein interaction docking (PPI‐Dock) simulations using MOE software with the S1–S2 and S3–S4 ECLs of VSD_I∼IV_ as the ligand‐binding sites. The top 300 minimized binding poses were prioritized based on the S values, which indicate the binding stability between channels and peptide ligands. As a result, both hSYN and BPP2 showed the best S scores (lowest in energy) in complex with the activated hCa_v_3.2 models, followed by the resting and inactivated models, while the complex with the original model was the least stable (Figure 4c). Notably, both hSYN and BPP2 were localized between ECL_I_ and ECL_IV_ in all of the top six complexes of the activated hCa_v_3.2 models (Figures 4d, S10). In the first (S score −135.87 kcal mol^−1^) and the third to sixth models, hSYN was mainly located in the ECL site, while the second model was bound to the TM site. Meanwhile, the first and third to fifth BPP2 models were entirely embedded in the TM site, while the second (S score −101.81 kcal mol^−1^) and sixth models were mainly bound to the ECL site. In contrast, in the other three complexes, the ligand‐binding sites showed considerable diversity, including TM site and outside the VSD (Figures S11–S13).

### Proposed Structure of the Activated hCa_v_3.2–hSYN Complex Obtained by MD Simulations

**Figure 5:**
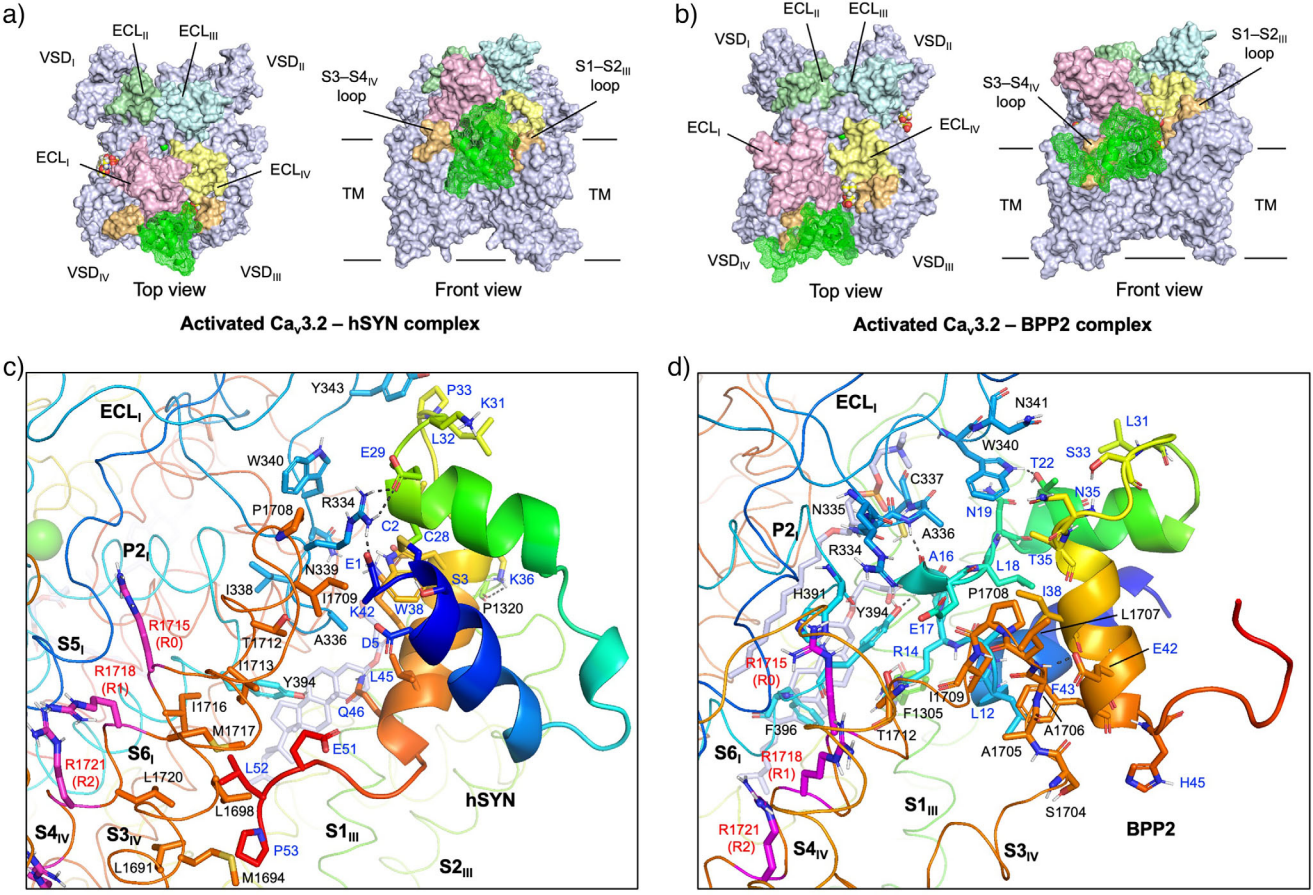
Proposed structures of hCa_v_3.2–hSYN and BPP2 complexes obtained by MD simulations. a,b) Structures of the hCa_v_3.2–hSYN and BPP2 complexes after 50 ns of simulations. hSYN and BPP2 are shown as green cartoon models with a mesh surface. Extracellular loop (ECL) domains I–IV and the S1–S2 and S3–S4 loops are highlighted in multi‐colors. c,d) Detailed views of the interactions between the activated hCa_v_3.2 channel and hSYN / BPP2 in (a) and (b). The side chains and selected main chains that contribute to the PPI between the channel (black letters) and ligands (blue letters) are highlighted with stick models, in which polar interactions are shown by black dotted lines.

Next, we performed molecular dynamics (MD) simulations to verify the stability of PPI‐docked models. Using YASARA software, the activated hCa_v_3.2–hSYN (rank 1) and BPP2 (rank 2) complexes as well as the apo channel were evaluated at a density of 0.997 g/mL, 310 K, and pH 7.4. In all three MD simulations during 50 ns, the “up” conformation of the gating charged residues of VSD_I∼IV_ and the open state of the inner channel gates were maintained (Figure S14). Both hSYN and BPP2 bound to similar sites across the domain‐swapped ECL_I,IV_ and TM sites, surrounded by the S1–S2_III_ (Leu1313–Gly1321) and S3–S4_IV_ (Met1703–Thr1712) loops (Figure 5a,b). In both complexes, the changes in conformational stability (C_α_ RMSD) and radiuses of gyration became smaller after 10 ns, suggesting that both of the two complexes were reliably stable (Figure S15 a,b). However, the hCa_v_3.2–BPP2 complex showed larger fluctuations (6.94 and 35.54 Å on average, respectively) than the hCa_v_3.2–hSYN complex (5.90 and 34.76 Å) and the apo channel (5.45 and 34.70 Å). With regard to the ligand site, hSYN moved less (the average C_α_ distances of each residue in 50 ns = 4.25 Å), than BPP2 (12.04 Å), especially in the α3–α4 loop and the C‐terminal tail after Glu42 (Figure S15c,d).

Detailed interactions of the hCa_v_3.2–hSYN and BPP2 complexes after 50 ns of simulation are shown in Figure 5c,d. hSYN interacted with ECL_I_ (Arg334∼Tyr344), P2–S6_I_ loop (Tyr394), S1–S2_III_ loop (Pro1320), and S3–S4_IV_ loop/helix (Leu1691∼Leu1720). In the complex structures recorded every 10 ns, hSYN continuously interacted with six residues, Arg334, Ala336, Trp340, Tyr343, Pro1708, and Thr1712 (Figure S16). Similarly, BPP2 bound to the hCa_v_3.2 channel at nearly the same positions as hSYN: ECL_I_ (Arg334∼Asn341), P2–S6_I_ loop (His391∼Phe396), S1_III_ (Phe1305), and S3–S4_IV_ loop (Ser1704∼Thr1712). BPP2 also continuously interacted with five residues in the 50 ns simulation: Arg334, Ala336, Cys337, Trp340, and Tyr394. Notably, these interaction sites and loop residues of hSYN and BPP2 are highly characteristic of T‐type channels (Ca_v_3.1∼3.3), which have little or no identity with N/P/Q/R‐type channels (Ca_v_2.1∼2.3). These results may explain why BPP2 had no significant effect on hCa_v_2.2 channel.

When we focused on peptide ligands, the residues interacting with the hCa_v_3.2 channel were highly different between hSYN and BPP2. In hSYN, the N‐terminal part of α1 helix, the latter half of α3 and α4 helices, and the C‐terminal tail were involved (Figure S16). Several electrostatic interactions were observed between hSYN and hCa_v_3.2 channel, including Glu1/Arg334, Glu29/Arg334, Lys36/Pro1320 (main C═O) (Figure 5c). In contrast, the interactions between BPP2 and hCa_v_3.2 channel included Thr22/Trp340, Ala16 (main C═O)/Ala336 (main NH), Ala16 (main NH)/Tyr394, and Glu42/A1706 (main NH) (Figure 5d). Among them, Glu1 and Lys36 in hSYN and Thr22 in BPP2 are unique residues, respectively. This difference may explain why the two ligands bound to hCa_v_3.2 in highly different orientations in the original PPI‐Dock models and after 50 ns of MD simulations.

When we focus on the voltage sensor domain residues, neither hSYN nor BPP2 directly interacted with R0 (Arg1715), R1 (Arg1718), or R2 (Arg1721) of S4 helix, but three C‐terminal residues of hSYN (Glu51, Leu52, and Pro53) hydrophobically bound to the nearby S3_IV_ and S4_IV_ helices (e.g., Met1694, Leu1698, Met1717) (Figure 5c). BPP2 also interacted with the S3–S4_IV_ loop at multiple sites in the α4 helix (Figure 5d). These unique interactions are expected to facilitate the functions of hSYN and BPP2 as voltage gating modulators in the T‐type Ca_v_ channels. Among the three subtypes of T‐type Ca_v_ channels, most of the residues that were assumed to interact with hSYN and BPP2 are conserved, but Arg334 is only in the hCa_v_3.2 channel (Figure S16). Although future electrophysiological evaluations are needed, our results suggest that ECL_I_ diversity may lead to Ca_v_ channel subtype specificity in shrew neurotoxins and their human analogues.

## Conclusion

Based on the finding that the exogenous shrew toxin BPPs paralyze prey, we investigated the human analogue hSYN, a non‐opioid fragment of PENK with unknown functions and target receptors in the central nervous system. Although hSYN exhibited similar activation activity for hCa_v_3.2 channel, it did not induce paralysis in mealworms, even at 10‐fold higher concentrations than BPP2. These results suggested that there are no direct relationships between the Ca_v_ channel actitation of BPP2 and paralytic activity in mealworms. Molecular docking and MD simulations identified key residues involved in interactions with the hCa_v_3.2 channel: Glu1 and Lys36 for hSYN and Thr22 for BPP2. We also confirmed that hSYN shares the same disulfide bond connectivity as BPP2 and adopts a highly similar conformation. ColabFold analysis suggested that the extended C‐terminal tail of synenkephalins does not affect their disulfide bond connectivity. On the other hand, the pre‐pro region of PENK (1–24) contains a conserved Cys residue found in human, rat, and *Blarina* shrew. Thus, different disulfide bond patterns could form in synenkephalins during the processing of this pre‐pro region. Although it remains unclear whether human synenkephalin exists as the Type‐I or Type‐II form in vivo, it is possible that the latter acts as a specific agonist for hCa_v_3.2 channel. Meanwhile, since high nanomolar concentrations (0.74–0.84 µM) of hSYN and BPP2 are required to hyperpolarize hCa_v_3.2 channel, synenkephalins could also bind to completely different, as‐yet unidentified, ion channels or receptors. Future research will aim to confirm the structures of other mammalian native synenkephalins in the central nervous system, and their target molecules using affinity pull‐down and photoaffinity labeling experiments, and to explore their involvement in pain transmission, in relation to the paralytic effects of BPPs.

It is believed that BPPs in shrews evolved as neurotoxins to paralyze invertebrate prey, and that their functions were enhanced through modifications of non‐toxic synenkephalins, which are ubiquitous in mammals. This accelerated venom evolution may explain the emergence of these molecules.^[^ ^76^, ^77^ ^]^ PENK, which contains sequences similar to hSYN and BPPs, is expressed in the submandibular glands of the European water shrew *Neomys fodiens* and the common shrew *Sorex araneus*.^[^ ^48^ ^]^ Although their synenkephalins have yet to be isolated or characterized, they may function as paralytic neurotoxins, similar to BPPs, helping these species efficiently capture specific prey and potentially offering an evolutionary advantage.^[^ ^13^, ^78^, ^79^ ^]^ Future studies will explore whether the convergent evolution observed in shrews represents a form of chemical evolution in mammalian venom. To achieve this, it will be necessary to study synenkephalin analogues in other insectivorous mammals, taking into account their feeding habits, relationships with natural enemies, and ecological roles.

The process by which BPPs are converted into mature peptides, consisting of 48–52 residues, in the submandibular glands remains unclear. One possibility is that synergistic effects from proteases, which are abundant in the same glands or saliva, contribute to this maturation. While these proteases are ineffective against invertebrates, a mouse‐lethal kallikrein‐like protease, blarina toxin (BLTX),^[^ ^80^ ^]^ and its non‐toxic homolog, blarinasin,^[^ ^81^ ^]^ have been identified in the same tissues as BPPs. Thus, co‐expression of these enzymes may lead to the diversification of toxic peptides through post‐translational modifications.^[^ ^20^, ^82^ ^]^ A similar process may occur in the brains and nervous systems of mammals, including humans. In this way, synergistic effects could facilitate the maturation of synenkephalins, leading to functional diversity, including interactions with unique target receptors.

Currently, it is difficult to predict from sequence data alone how human synenkephalin is processed in vivo and which receptors in the brain it interacts with. Research on the endogenous functions of orphan neuropeptides (including precursor peptides) and orphan receptors is still ongoing.^[^ ^83^, ^84^ ^]^ As demonstrated in this study, focusing on the evolutionary context and functional diversity of endogenous and exogenous peptides may uncover new functional sites and signal transduction pathways in the nervous system. In the future, we hope to conduct interactome analyses that integrate biological activity, conformation, interaction sites, localization, and intracellular signaling of ligands, to further investigate the functions and diversity of neuropeptides as both endogenous ligands and natural venoms.

## Conflict of Interests

The authors declare no conflict of interest.

## Supporting information



Supporting Information

## Data Availability

The data that support the findings of this study are available in the supplementary material of this article.
