# Supplementary material for: Hyperpolarization Modulation of the T‐Type hCav3.2 Channel by Human Synenkephalin [1–53], a Shrew Neurotoxin Analogue without Paralytic Effects
Source: Angew Chem Int Ed Engl. 2025 May 2;64(27):e202503891. doi: 10.1002/anie.202503891 (PMC12207360; doi:10.1002/anie.202503891)
Supplement: Supplementary file 1 — Supporting Information [file ANIE-64-e202503891-s001.pdf]

Supporting Information

©Wiley-VCH 2025

69451 Weinheim, Germany

# Hyperpolarization Modulation of the T-type $hCa_v3.2$ Channel by Human Synenkephalin [1–53], a Shrew Neurotoxin Analogue without Paralytic Effects

Ryo Fukuoka, Yusuke Yano, Nozomi Hara, Chihiro Sadamoto, Andres D. Maturana, and Masaki Kita *

**Abstract:** Mammalian secreted venoms mainly consist of peptides and proteases used for defense or predation. *Blarina* paralytic peptides (BPPs), mealworm-targeting neurotoxins from shrew, are very similar to human synenkephalin. This peptide is released from proenkephalin in the brain along with opioid peptides that mediate analgesic and antidepressant effects, though its physiological function is unclear. Here we synthesized and characterized human synenkephalin [1–53] (hSYN), and reveal its disulfide bond connectivity. Similar to BPP2, hSYN caused a hyperpolarizing shift in the human T-type voltage-gated calcium channel ($hCa_v3.2$) at 0.74 μM, but did not paralyze mealworms. Molecular docking and molecular dynamics simulations showed that hSYN and BPP2 interact with $hCa_v3.2$ channel differently, due to differences in polar residues. Since $Ca_v3.2$ channel regulates neuronal excitability and is implicated in conditions like autism and epilepsy, our findings on hSYN could provide insights into the channel gating and agonistic mechanisms, along with potential pathways for developing treatments for neurological disorders.

DOI: 10.1002/anie.2021XXXXX

## Contents

| | |
|---|---|
| Supporting figures | S1~S17 |
| Materials and methods | S18~S25 |
| Supporting references | S26 |

**a**

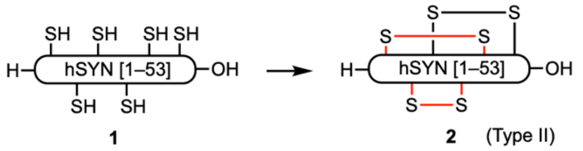


| entry | oxidant | solvent | pH | temp. (°C) | yield (%) |
|---|---|---|---|---|---|
| 1 | Air | 0.1 M $NH_4HCO_3$ | 8.0 | 25 | – [a] |
| 2 | DMSO | 40% MeCN aq. | 6.0 | 25 | – [a] |
| 3 | cystein / cystine | 0.1 M $NH_4OAc$, 20% EtOH | 8.5 | 37 | 65 |

[a] Not detected.

**b**

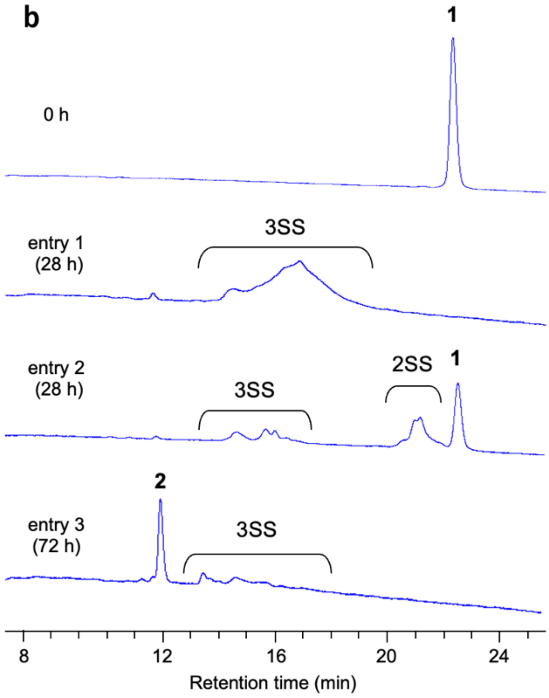


**c**

**d**

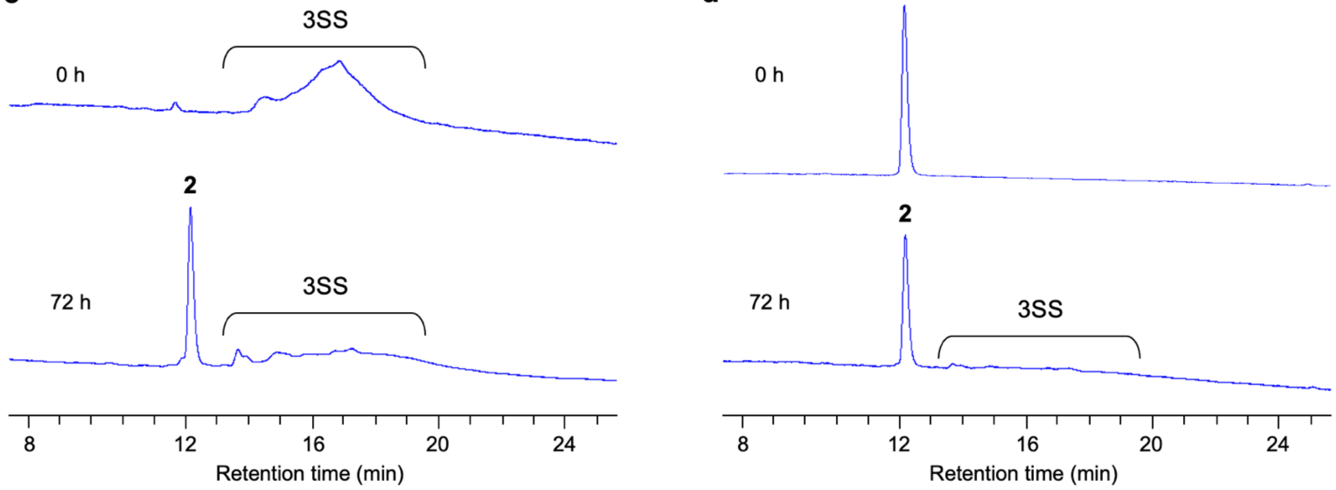


**Figure S1.** Preparation of Type-II hSYN [1–53] (**2**) from linear peptide **1**. (a) Summary of the oxidation and folding reaction conditions. (b) Analysis of the refolded products in (a). HPLC conditions: Develosil ODS HG-5 (5 µm, ϕ 4.6 mm × 250 mm), 30% to 60% aq. MeCN / 0.1% TFA gradient for 30 min, flow: 1.0 ml/min, 23 °C, monitoring at UV 214 nm. On entry 3, desired **2** ($t_R$ = 12.0 min) was obtained in 65% yield along with trace amount of misfolded 3SS analogues (see Scheme 1). (c, d) Analysis of the refolded hSYN analogues. In (c), misfolded hSYN analogues in entry 1 ($t_R$ 13.5–19.5 min) in (a) (top) was treated with the cysteine-cystine refolding condition for 72 h (bottom) as with the entry 3 in (a). In (d), purified **2** (top) was further treated with the same refolding condition for 72 h (bottom).

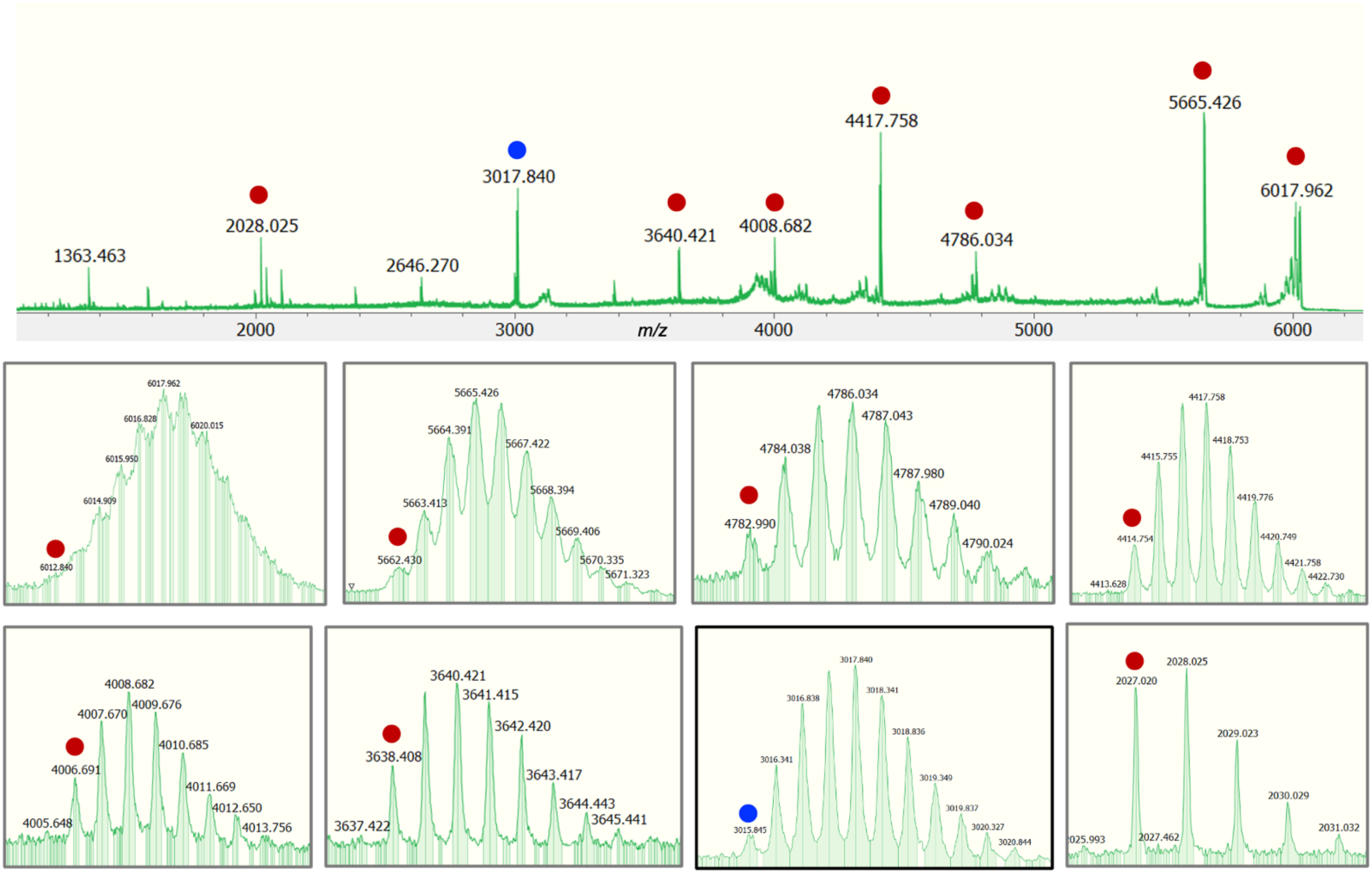


1 10 20 30 40 50

ECSQDCATCSYRLVRPADINFLACVMECEGKLPSLKIWETCKELLQLSKPELP

| Position | Numbers of SS bonds | $[M+H]^+_{calc.}$ | $[M+H]^+_{obs.}$ | Remarks |
|---|---|---|---|---|
| [37–53] | 0 | 2027.1 | 2027.0 | Cys41: free SH |
| [1–53] | 3 | 3016.0 | 3015.8 | $[M+2H]^{2+}$, 2 × $H_2O$ adduct |
| [1–12, 16–36] | 2 | 3638.6 | 3638.4 | Cys9: free SH |
| [1–36] | 2 | 4006.8 | 4006.7 | 1 × $H_2O$ adduct |
| [1–12, 16–42] | 3 | 4414.9 | 4414.8 | 1 × $H_2O$ adduct |
| [1–42] | 3 | 4783.2 | 4783.0 | 2 × $H_2O$ adduct |
| [1–12, 16–53] | 3 | 5662.6 | 5662.4 | 1 × $H_2O$ adduct |
| [1–53] | 3 | 6012.9 | 6012.8 | 1 × $H_2O$ adduct |

**Figure S2.** Intact tryptic digestion of Type-II hSYN [1–53] (**2**) without disulfide bond reduction. MALDI-TOF MS of digested peptide mixture (top), sequence of **2** (middle), and assigned monoisotopic ion data (bottom) are shown.

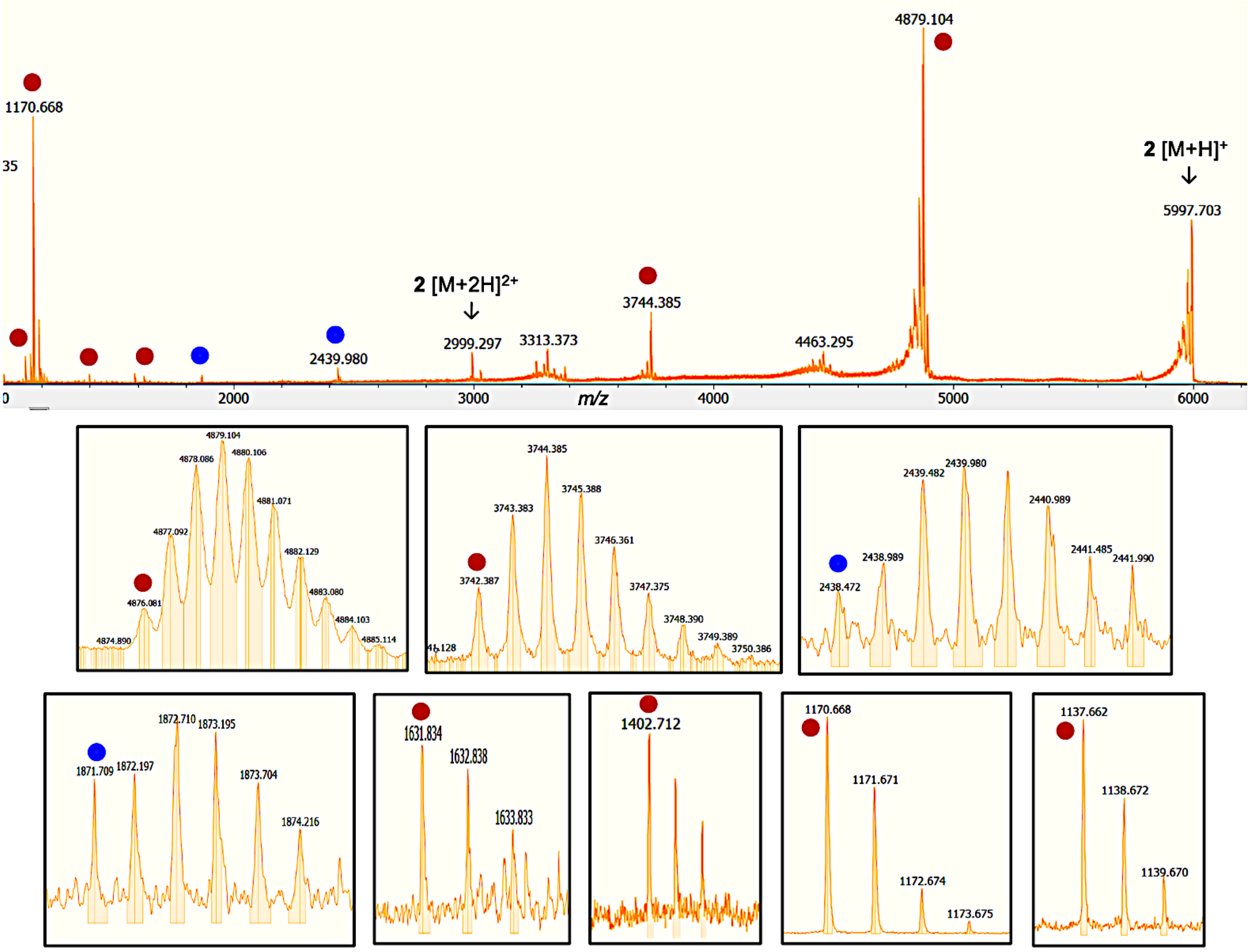


1 10 20 30 40 50

<u>E</u>CSQ<u>D</u>CATCSYRLVRPA<u>D</u>INFLACVM<u>E</u>C<u>E</u>GKLPSLKIW<u>E</u>TCK<u>E</u>LLQLSKP<u>E</u>LP

| Position | Numbers of SS bonds | $[M+H]^+$ calc. | $[M+H]^+$ obs. | Remarks |
|---|---|---|---|---|
| [44–53] | 0 | 1137.7 | 1137.7 | |
| [30–39] | 0 | 1170.7 | 1170.7 | |
| [28–39] | 0 | 1402.7 | 1402.7 | Cys28: free SH |
| [30–43] | 0 | 1631.9 | 1631.8 | Cys41: free SH |
| [1–29, 40–43] | 3 | 1871.8 | 1871.7 | $[M+2H]^{2+}$ |
| [1–43] | 3 | 2438.7 | 2438.5 | $[M+2H]^{2+}$ |
| [1–29, 40–43] | 3 | 3742.5 | 3742.4 | |
| [1–43] | 3 | 4876.3 | 4876.1 | |

**Figure S3.** Intact Glu-C digestion of Type-II hSYN [1–53] (**2**) without disulfide bond reduction. MALDI-TOF MS of digested peptide mixture (top), sequence of **2** (middle), and assigned monoisotopic ion data (bottom) are shown.

**a**

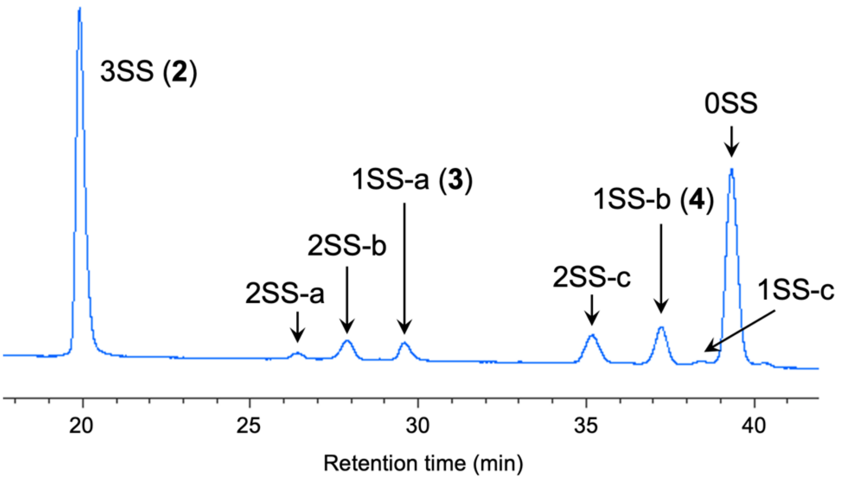


**b**

**3 :** EC$^{2}$(Cam)SQDC$^{6}$(Nem)ATC$^{9}$(Nem)SYRLVRPADINFLAC$^{24}$(Nem)VMEC$^{28}$(Cam)EGKLPSLKIWETC$^{41}$(Nem)KELLQLSKPELP

| position | $[M+H]^{+}_{Calc.}$ | $[M+H]^{+}_{Obs.}$ | Identified fragments |
|---|---|---|---|
| [1-12] | 1672.6 | 1672.9 | EC$^{2}$(Cam)SQDC$^{6}$(Nem)ATC$^{9}$(Nem)SYR |
| [13-31] | 2290.1 | 2290.4 | LVRPADINFLAC$^{24}$(Nem)VMEC$^{28}$(Cam)EGK |
| [32-42] | 1442.8 | 1443.0 | LPSLKIWETC$^{41}$(Nem)K |
| [43-53] | 1266.7 | 1266.9 | ELLQLSKPELP |

**4 :** EC$^{2}$(Nem)SQDC$^{6}$(Cam)ATC$^{9}$(Nem)SYRLVRPADINFLAC$^{24}$(Cam)VMEC$^{28}$(Nem)EGKLPSLKIWETC$^{41}$(Nem)KELLQLSKPELP

| position | $[M+H]^{+}_{Calc.}$ | $[M+H]^{+}_{Obs.}$ | Identified fragments |
|---|---|---|---|
| [1-12] | 1672.6 | 1672.9 | EC$^{2}$(Nem)SQDC$^{6}$(Cam)ATC$^{9}$(Nem)SYR |
| [13-31] | 2290.1 | 2290.5 | LVRPADINFLAC$^{24}$(Cam)VMEC$^{28}$(Nem)EGK |
| [32-42] | 1442.8 | 1443.1 | LPSLKIWETC$^{41}$(Nem)K |
| [43-53] | 1266.7 | 1267.0 | ELLQLSKPELP |

**Figure S4.** Disulfide bond connectivity analysis of Type II hSYN [1–53] (**2**). (a) HPLC chromatogram of the partially reduced and NEM-alkylated peptides from **2**. HPLC conditions: Develosil ODS HG-5 (5 µm, ϕ 4.6 mm × 250 mm), 20% aq. MeCN / 0.1% TFA, 5 min; 20–50% aq. MeCN / 0.1% TFA linear gradient for 60 min, flow: 1.0 mL/min, 25 °C, monitoring at UV 214 nm. (b) Structures of the 1SS analogues **3** and **4**. After HPLC separation as shown in (a), the remaining disulfide bonds in **3** and **4** were reduced with DTT and alkylated with IAM. Assigned monoisotopic ion data and structures of the tryptic peptides by MALDI-TOF MS are shown.

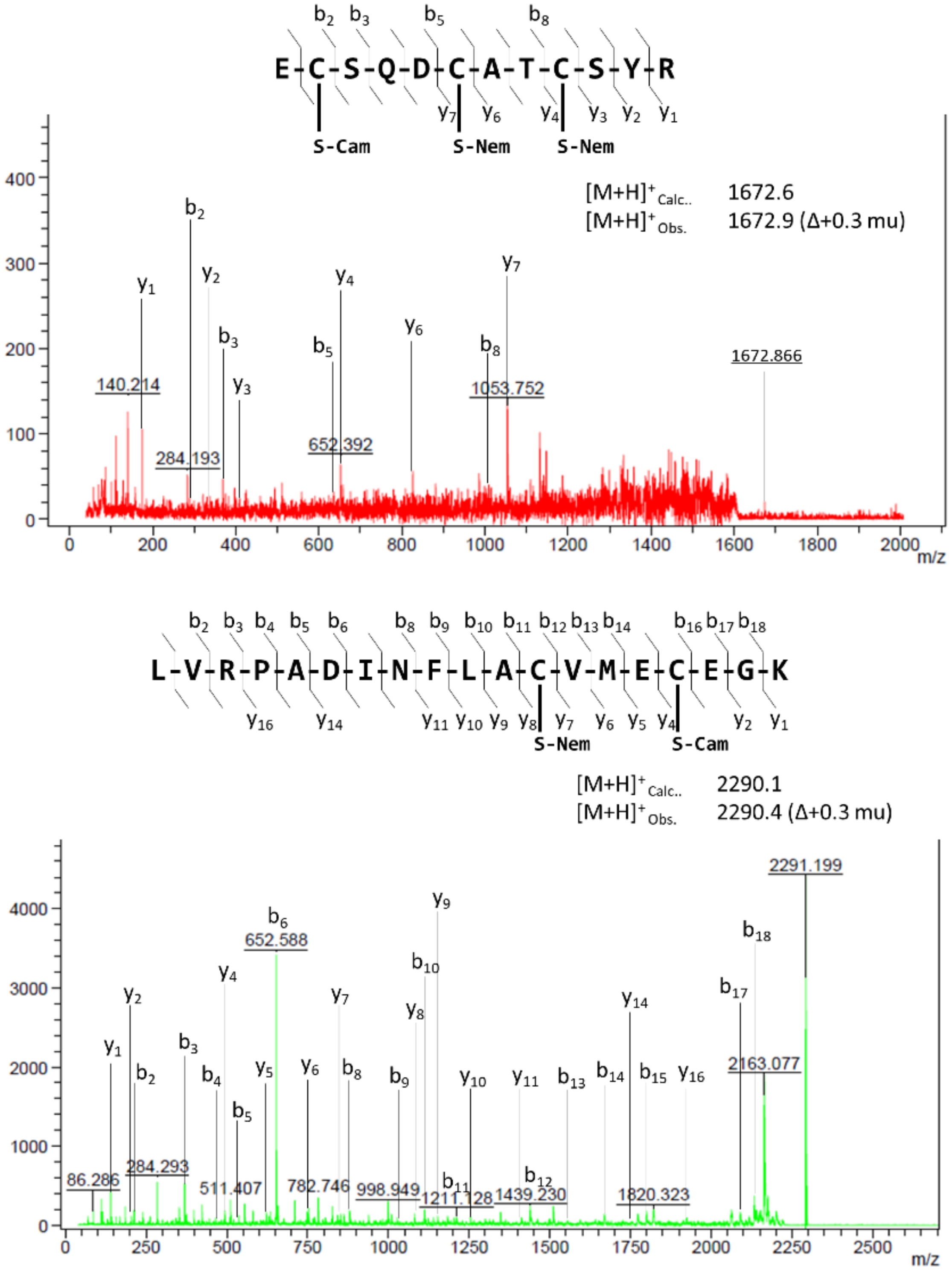


**Figure S5.** MALDI MS/MS analysis of digested peptides from the 1SS analogue **3**. For detail, see Figure S4.

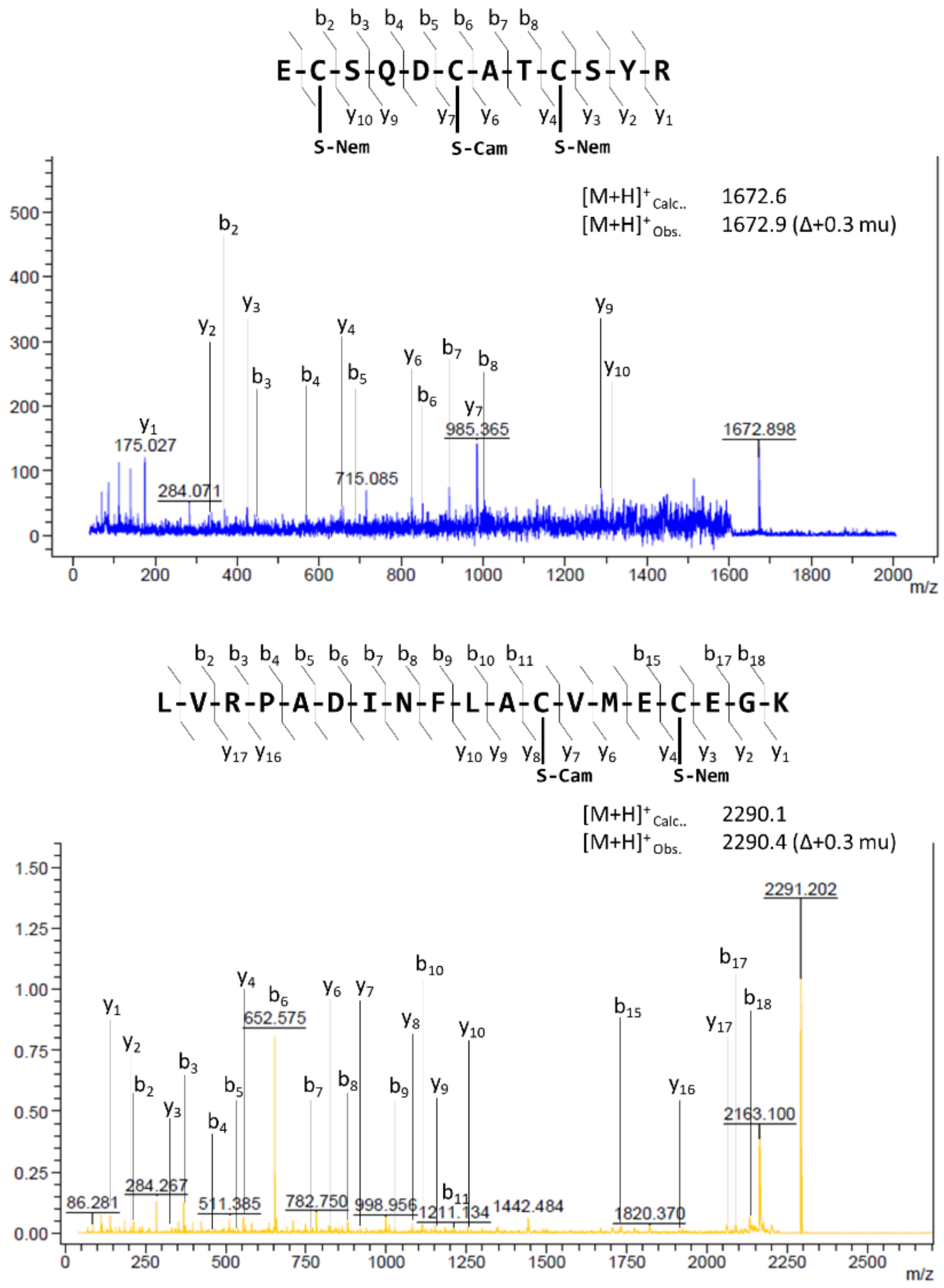


**Figure S6.** MALDI MS/MS analysis of digested peptides from the 1SS analogue **4**. For detail, see Figure S4.

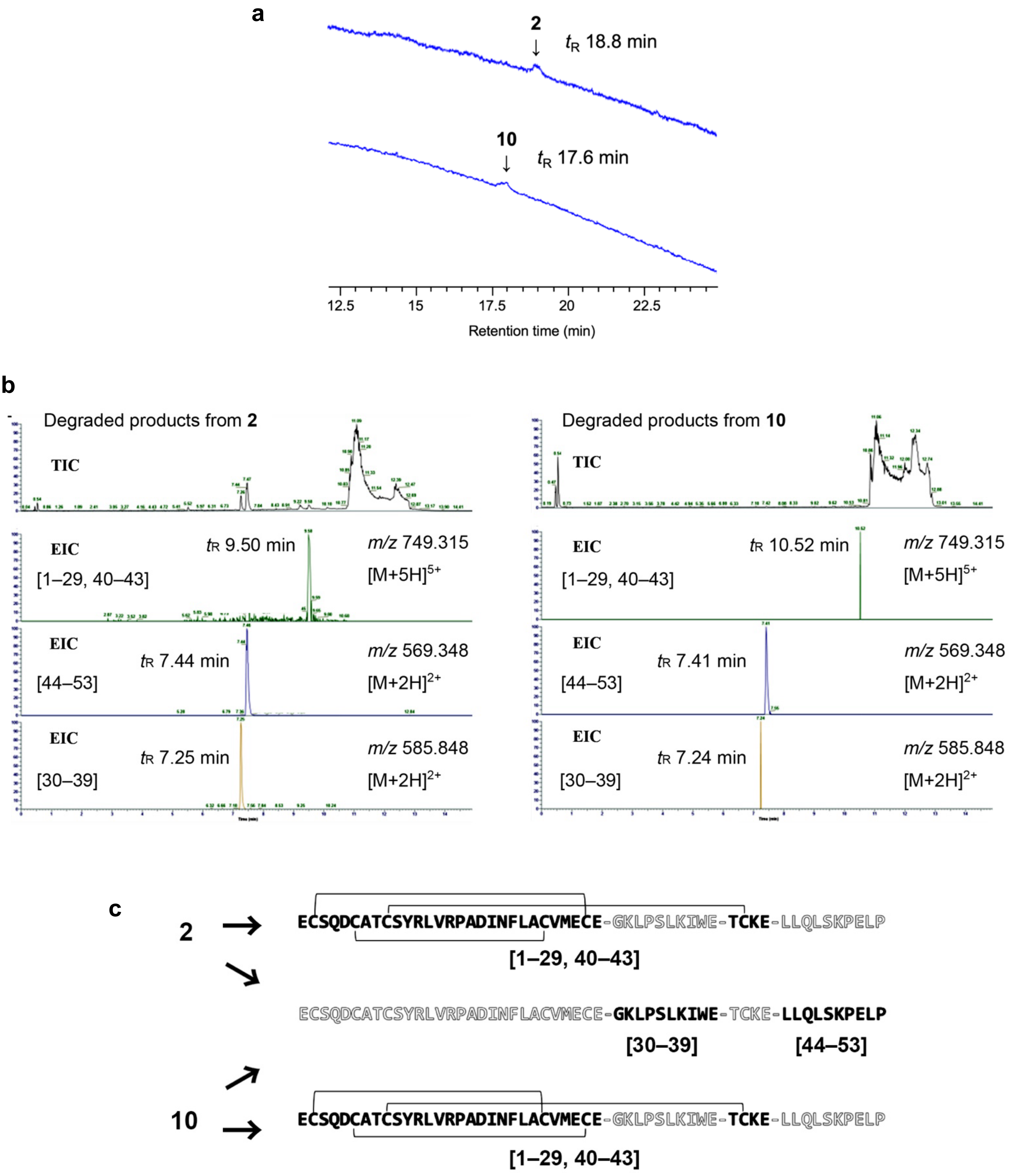


**Figure S7.** Comparison of Type-II hSYN [1–53] (**2**) and Type-I hSYN [1–53] (**10**). (a) HPLC analysis of original **2** and **10**. Conditions: Agilent ZORBAX SB-C18 (3.5 µm, ϕ 0.5 mm × 150 mm), temp. 40 ºC, 25% for 10 min and 25–45% linear gradient for 20 min, flow rate 16 µL/min, UV215 nm (A; 0.1% aq. TFA, B; 0.08% TFA/MeCN). (b) LC-MS analysis of the degraded products obtained from **2** and **10** by Glu-C digestion without disulfide bond reduction. (c) Proposed structures of the degraded products in (b).

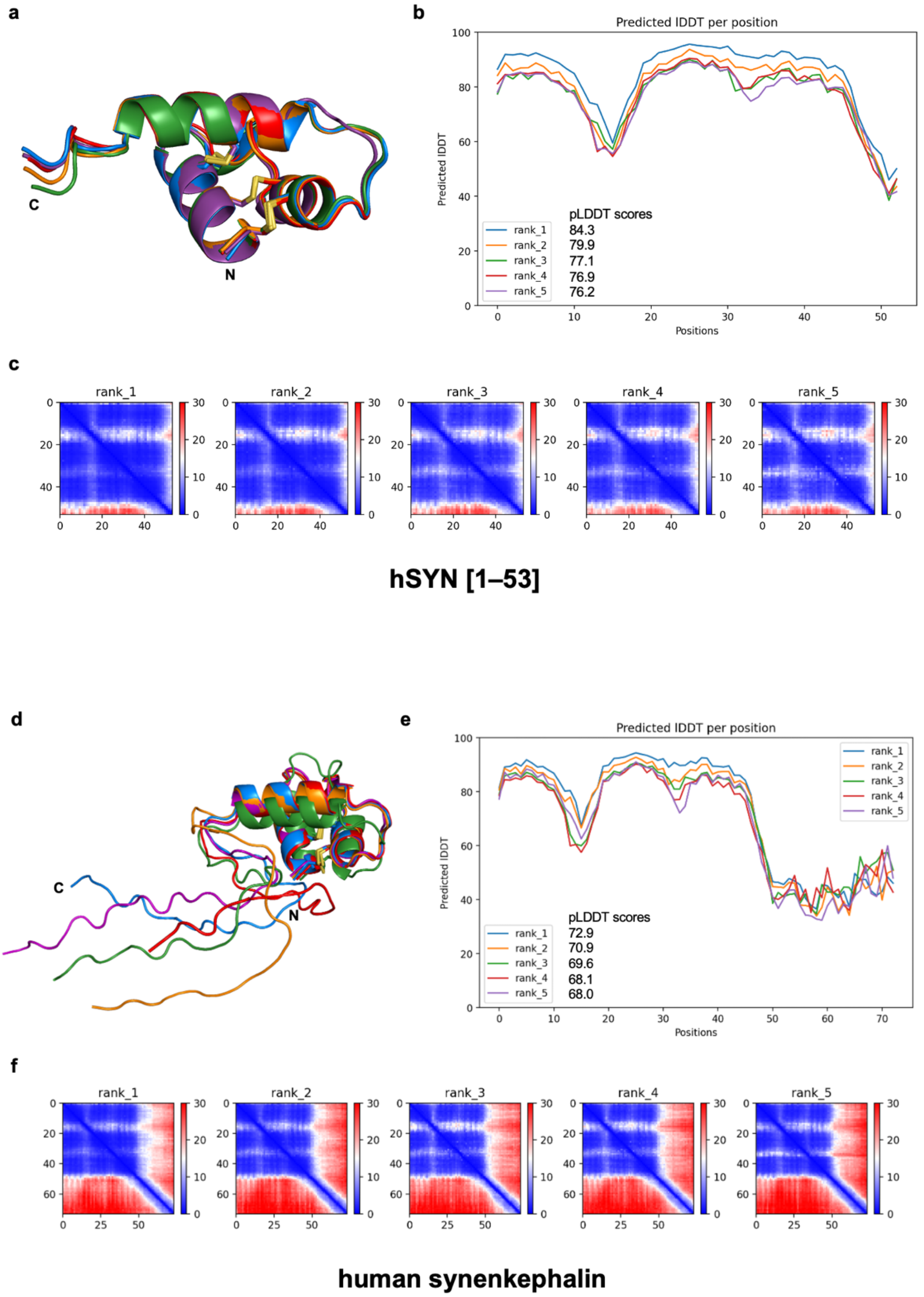


**Figure S8.** Structure predictions of hSYN [1–53] and human synenkephalin using ColabFold. (a) and (d): Alignment of top five models. Disulfide bonds are shown in stick models. (b) and (e): Predicted lDDT per residues of the top five models. (c) and (f): Calculated predicted aligned errors of the five models.

**a**

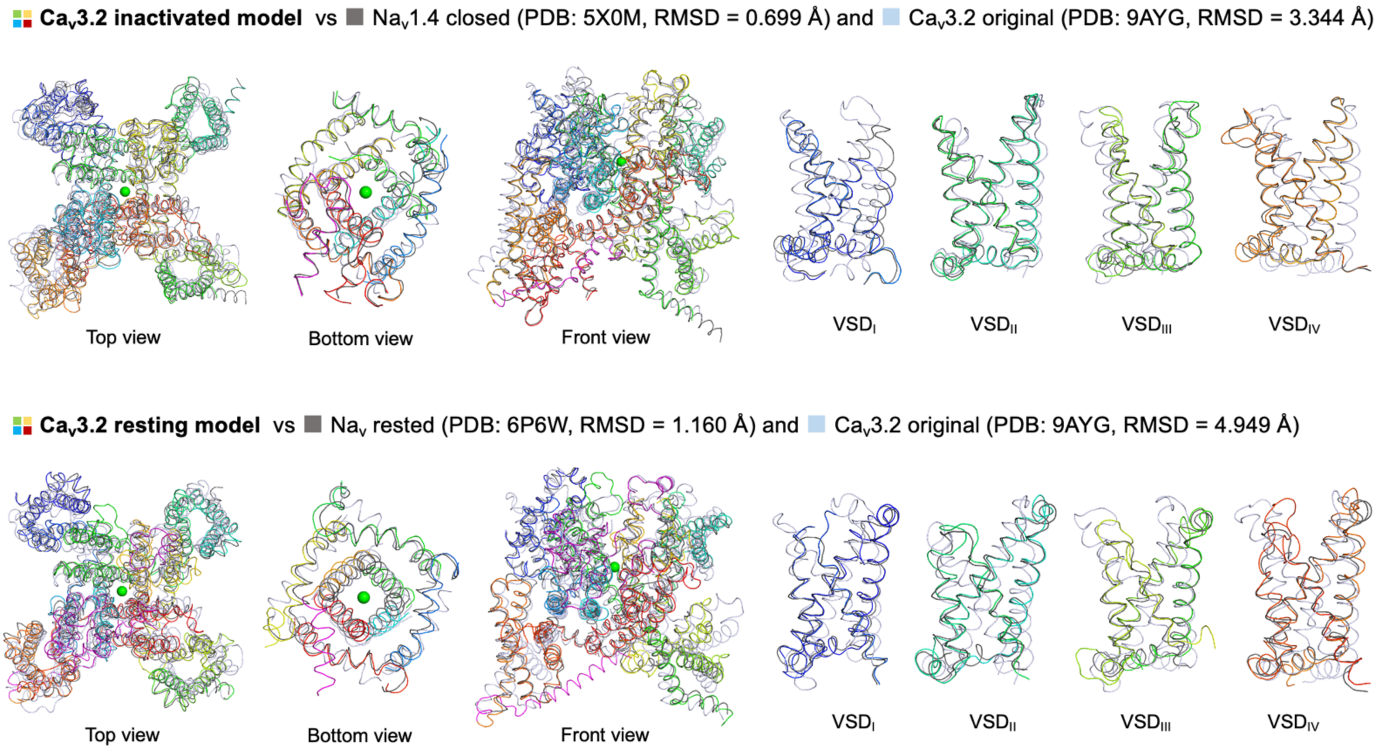


**b**

| templates | homology models | | | Ca$_v$3.2 |
|---|---|---|---|---|
| | activated | inactivated | rested | [9AYG] |
| Na$_v$1.4 open [6AGF] | 0.669 | 3.062 | 5.357 | 2.396 |
| Na$_v$1.4 close [5X0M] | 3.123 | 0.699 | 5.158 | 3.126 |
| Na$_v$ rested [6P6W] | 4.807 | 4.176 | 1.160 | 3.366 |
| Ca$_v$3.2 [9AYG] | 2.868 | 3.344 | 4.949 | – |

RMSD (Å)

**c**

R0 R1 R2 R3/K3 R4/K4 R4+1 K5 R5+1

VSD$_I$ AIRTVRVLRPLRAINRVPSM

VSD$_{II}$ VLRTFRLLRVLKLVRFLPAL

VSD$_{III}$ VLRVLRLLRTLRPLRVISRA

VSD$_{IV}$ IIRIMRVLRIARVLKLLKMA

**d**

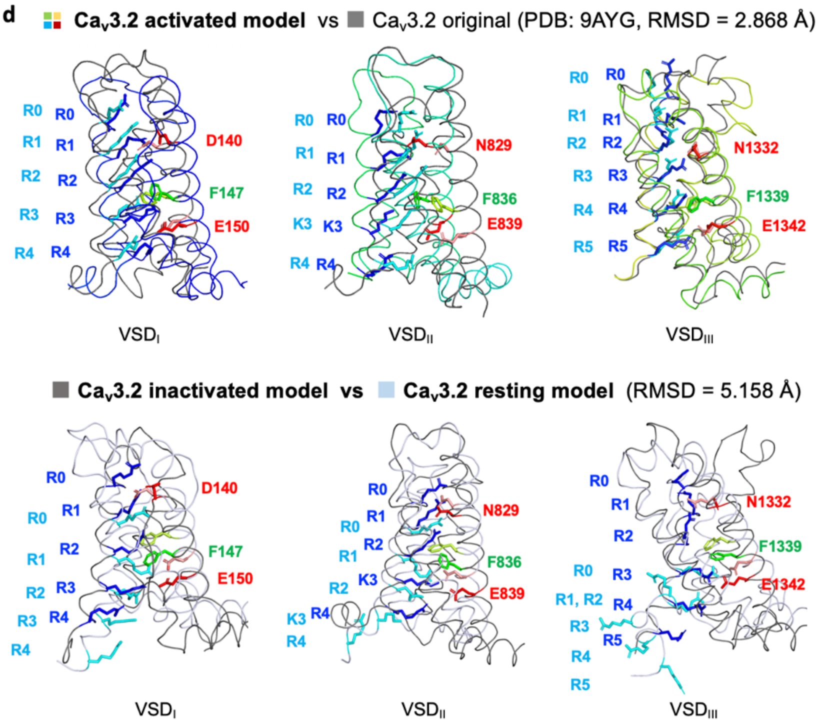


**Figure S9.** Structures of hCa$_v$3.2 channel models obtained by homology modeling. (a) Comparison of inactivated / resting hCa$_v$3.2 models with Na$_v$1.4 closed / Na$_v$ rested structures and the hCa$_v$3.2 original structure. The missing S3–S4 linkers in the hCa$_v$3.2 cyro-EM structure are shown in magenta. On the resting model, the ECL domain constructed by the replacement with that of hCa$_v$3.2 original structure is shown in purple. (b) RMSD values of the homology models compared with the template Na$_v$ channels and hCa$_v$3.2 original structures. Each PDB ID is shown in square brackets. (c) S4 helix sequences on the VSD$_{I\sim IV}$ of hCa$_v$3.2 channel. (d) Comparison of VSD$_{I\sim III}$ structures. Characteristic gating positive-charged residues at the S4 helix and their facing residues at the adjacent S2 and S3 helices are highlighted in stick models.

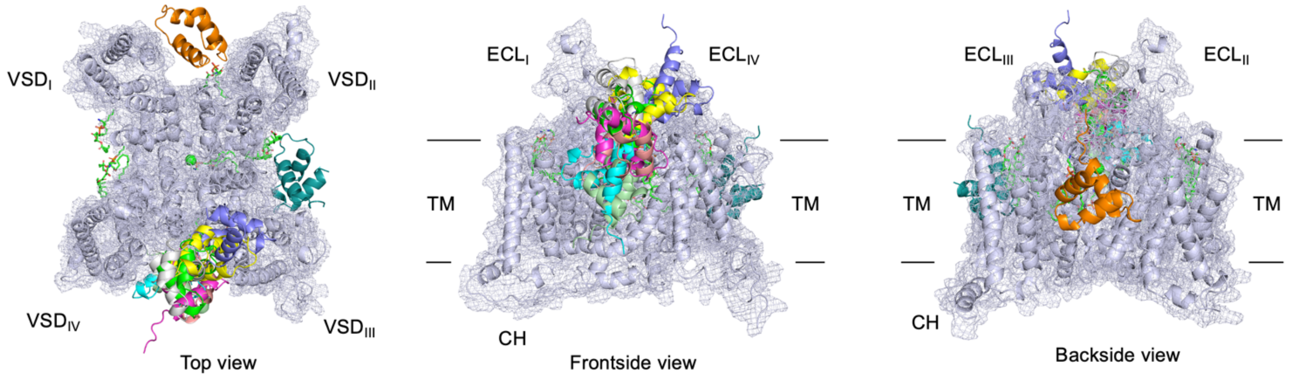


**Activated Ca$_v$3.2 – hSYN complex**

| No. | color | Docking score (kcal/mol) | Relative energy (kcal/mol) | Binding site (mainly) |
|---|---|---|---|---|
| 1 | green | -135.87 | 0.00 | ECL1-4 |
| 2 | cyan | -82.73 | 53.13 | TM |
| 3 | magenta | -76.46 | 59.41 | ECL1-4 |
| 4 | yellow | -64.15 | 71.72 | ECL1-4 |
| 5 | salmon | -63.77 | 72.10 | ECL1-4 |
| 6 | grey | -63.55 | 72.31 | ECL1-4 |
| 7 | skyblue | -60.29 | 75.58 | ECL outside |
| 8 | orange | -58.16 | 77.71 | TM |
| 9 | palegreen | -56.93 | 78.94 | TM |
| 10 | deepteel | -56.89 | 78.98 | TM |

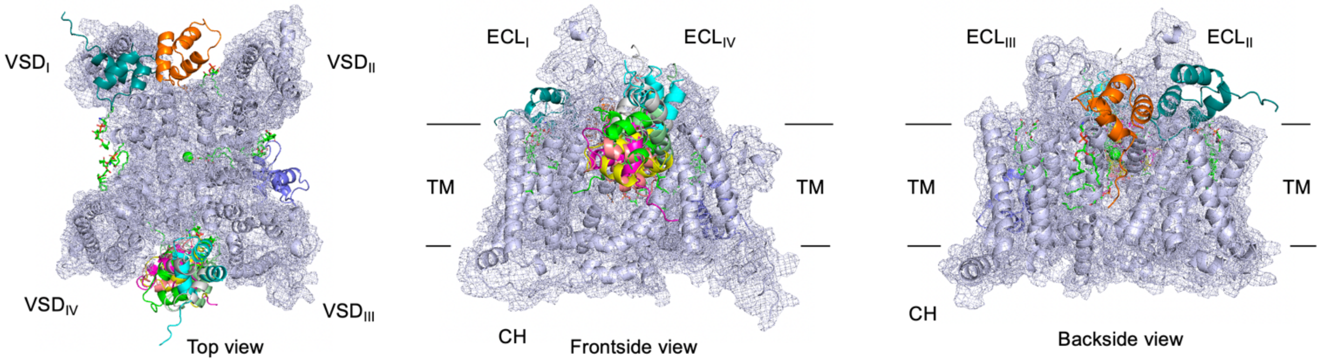


**Activated Ca$_v$3.2 – BPP2 complex**

| No. | color | Docking score (kcal/mol) | Relative energy (kcal/mol) | Binding site (mainly) |
|---|---|---|---|---|
| 1 | green | -150.08 | 0.00 | TM |
| 2 | cyan | -101.81 | 48.26 | ECL1-4 |
| 3 | magenta | -87.89 | 62.19 | TM |
| 4 | yellow | -83.90 | 66.18 | TM |
| 5 | salmon | -78.21 | 71.87 | TM |
| 6 | grey | -67.66 | 82.41 | ECL1-4 |
| 7 | skyblue | -67.05 | 83.02 | TM |
| 8 | orange | -64.84 | 85.24 | ECL2-3 |
| 9 | palegreen | -64.02 | 86.05 | ECL1-4 |
| 10 | deepteel | -62.94 | 87.13 | VSD1 |

**Figure S10.** Superimposed structures of the top 10 models of the activated hCa$_V$3.2–hSYN [1–53] and BPP2 complexes with the lowest docking scores. The rank 1 model (green) for hSYN and the rank 2 model (cyan) for BPP2 highlighted in pink boxes were used for further MD simulations.

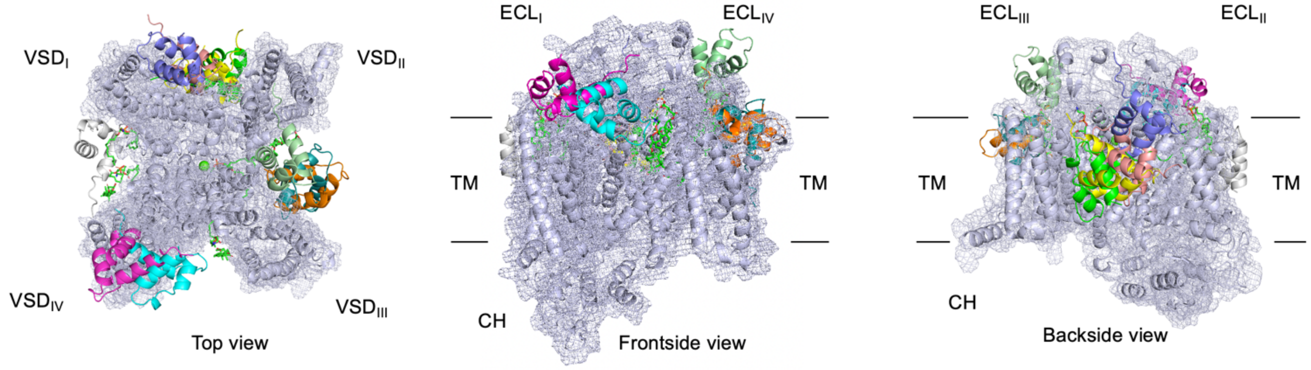


**Inactivated $Ca_v$3.2 – hSYN complex**

| No. | color | Docking score (kcal/mol) | Relative energy (kcal/mol) | Binding site (mainly) |
|---|---|---|---|---|
| 1 | green | -65.52 | 0.00 | TM |
| 2 | cyan | -64.17 | 1.35 | ECL1-4 |
| 3 | magenta | -63.25 | 2.27 | VSD4 |
| 4 | yellow | -61.89 | 3.63 | TM |
| 5 | salmon | -61.32 | 4.20 | TM |
| 6 | grey | -59.94 | 5.58 | TM |
| 7 | skyblue | -59.29 | 6.23 | ECL2-3 |
| 8 | orange | -57.72 | 7.80 | TM |
| 9 | palegreen | -57.48 | 8.04 | ECL3-4 |
| 10 | deepteel | -56.82 | 8.70 | TM |

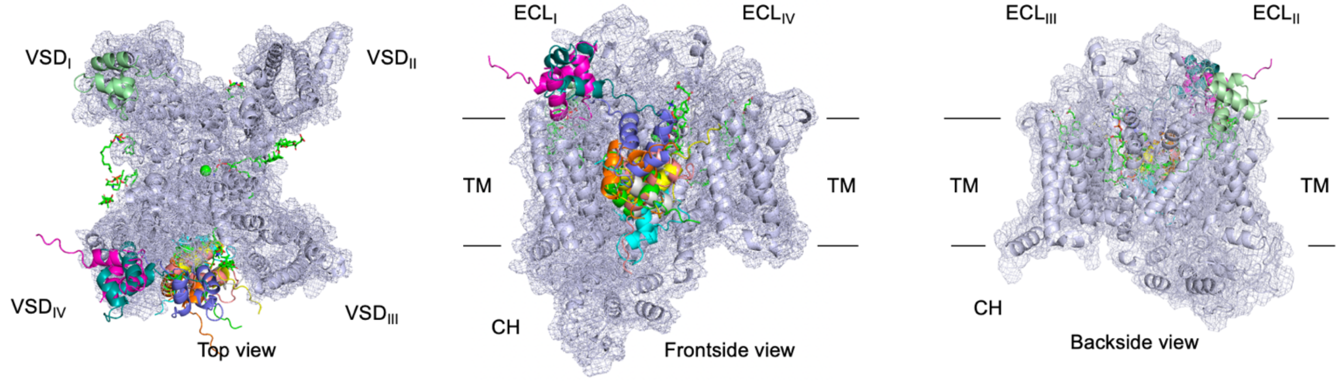


**Inactivated $Ca_v$3.2 – BPP2 complex**

| No. | color | Docking score (kcal/mol) | Relative energy (kcal/mol) | Binding site (mainly) |
|---|---|---|---|---|
| 1 | green | -74.52 | 0.00 | TM |
| 2 | cyan | -69.28 | 5.23 | TM |
| 3 | magenta | -62.90 | 11.61 | VSD4 |
| 4 | yellow | -61.36 | 13.16 | TM |
| 5 | salmon | -60.61 | 13.91 | TM |
| 6 | grey | -59.64 | 14.88 | TM |
| 7 | skyblue | -59.19 | 15.32 | TM |
| 8 | orange | -58.98 | 15.54 | TM |
| 9 | palegreen | -57.46 | 17.06 | VSD1 |
| 10 | deepteel | -56.26 | 18.26 | VSD4 |

**Figure S11.** Superimposed structures of the top 10 models of the inactivated h$Ca_v$3.2–hSYN [1–53] and BPP2 complexes with the lowest docking scores.

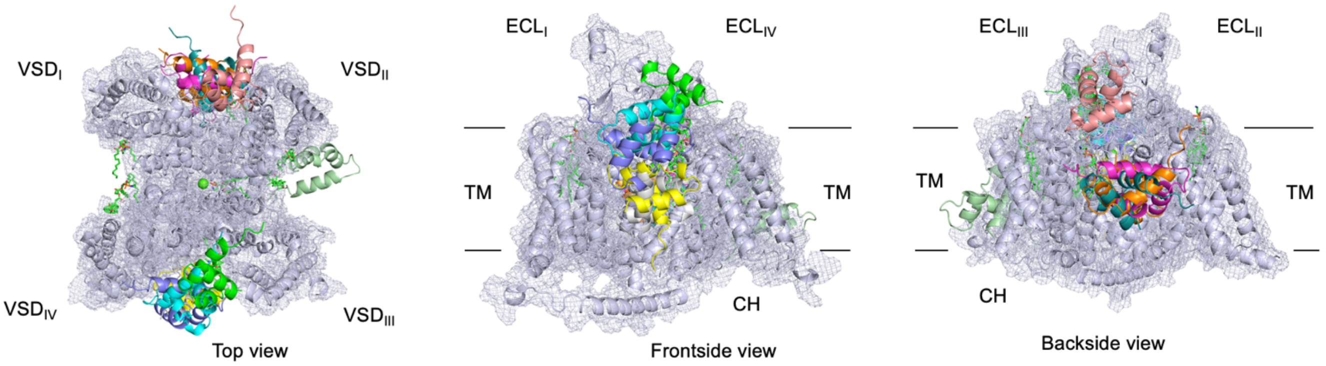


**Resting Ca$_v$3.2 – hSYN complex**

| No. | color | Docking score (kcal/mol) | Relative energy (kcal/mol) | Binding site (mainly) |
|---|---|---|---|---|
| 1 | green | -97.71 | 0.00 | ECL1-4 |
| 2 | cyan | -89.23 | 8.48 | ECL1-4 |
| 3 | magenta | -59.28 | 38.43 | TM |
| 4 | yellow | -57.69 | 40.03 | TM |
| 5 | salmon | -57.33 | 40.38 | ECL2-3 |
| 6 | grey | -56.56 | 41.15 | TM |
| 7 | skyblue | -55.03 | 42.68 | TM |
| 8 | orange | -54.64 | 43.08 | TM |
| 9 | palegreen | -54.45 | 43.27 | TM |
| 10 | deepteel | -54.29 | 43.42 | TM |

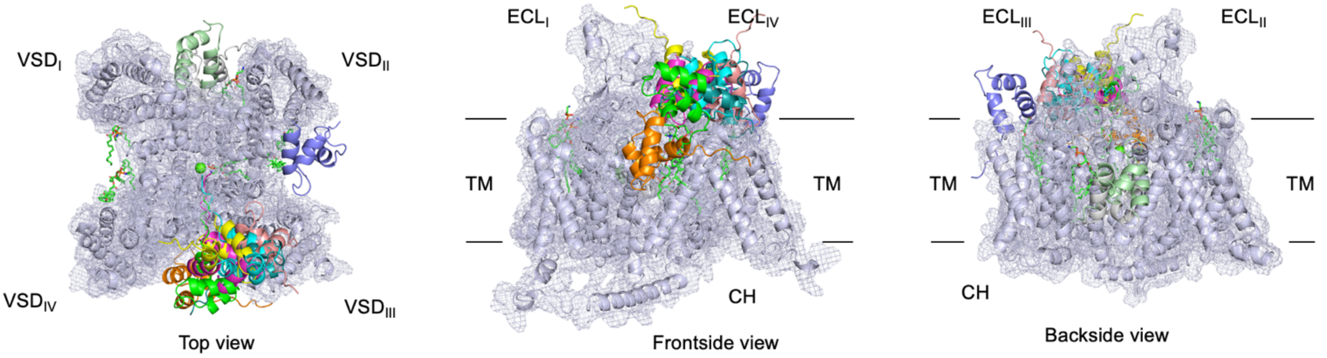


**Resting Ca$_v$3.2 – BPP2 complex**

| No. | color | Docking score (kcal/mol) | Relative energy (kcal/mol) | Binding site (mainly) |
|---|---|---|---|---|
| 1 | green | -114.10 | 0.00 | ECL1-4 |
| 2 | cyan | -81.48 | 32.62 | VSD3 |
| 3 | magenta | -80.45 | 33.65 | ECL1-4 |
| 4 | yellow | -74.00 | 40.09 | ECL1-4 |
| 5 | salmon | -70.78 | 43.31 | VSD3 |
| 6 | grey | -67.96 | 46.14 | TM |
| 7 | skyblue | -63.82 | 50.28 | ECL3-4 |
| 8 | orange | -61.84 | 52.26 | TM |
| 9 | palegreen | -60.45 | 53.65 | TM |
| 10 | deepteel | -59.91 | 54.19 | VSD3 |

**Figure S12.** Superimposed structures of the top 10 models of the resting hCa$_v$3.2–hSYN [1–53] and BPP2 complexes with the lowest docking scores.

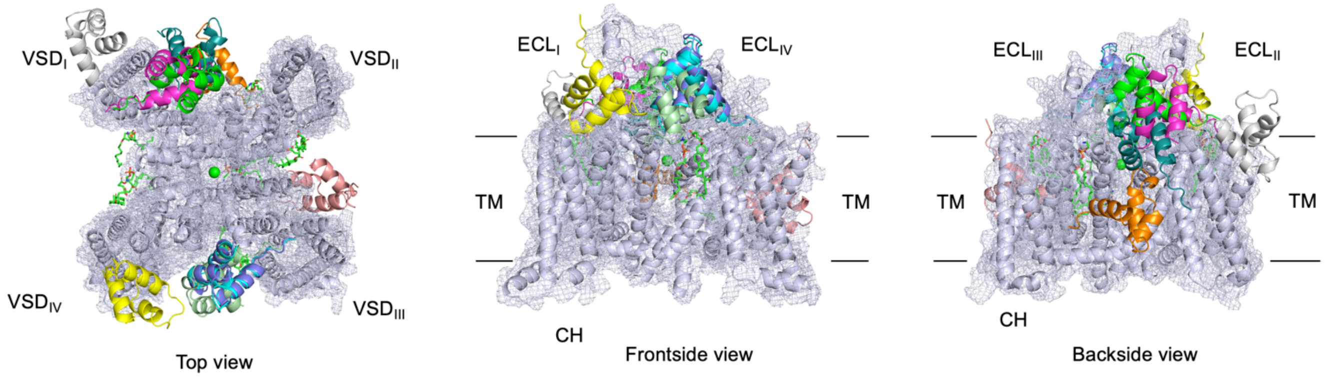


**Original Ca$_v$3.2 – hSYN complex**

| No. | color | Docking score (kcal/mol) | Relative energy (kcal/mol) | Binding site (mainly) |
|---|---|---|---|---|
| 1 | green | -55.61 | 0.00 | ECL2-3 |
| 2 | cyan | -55.37 | 0.24 | ECL1-4 |
| 3 | magenta | -55.24 | 0.36 | ECL2-3 |
| 4 | yellow | -53.97 | 1.64 | VSD4 |
| 5 | sarmon | -53.76 | 1.84 | TM |
| 6 | grey | -53.42 | 2.19 | VSD1 |
| 7 | skyblue | -53.14 | 2.47 | ECL1-4 |
| 8 | orange | -52.89 | 2.72 | TM |
| 9 | palegreen | -51.77 | 3.84 | ECL1-4 |
| 10 | deepteel | -51.56 | 4.05 | ECL2-3 |

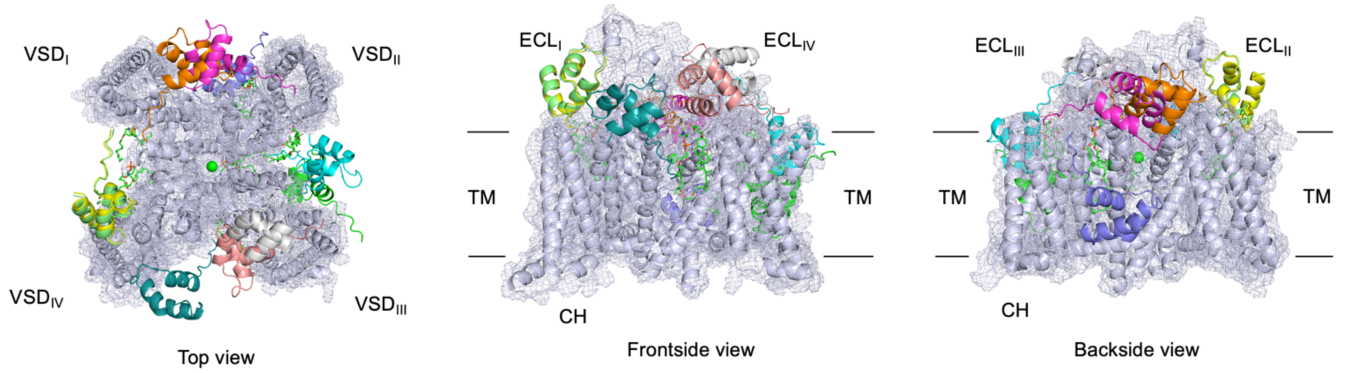


**Original Ca$_v$3.2 – BPP2 complex**

| No. | color | Docking score (kcal/mol) | Relative energy (kcal/mol) | Binding site (mainly) |
|---|---|---|---|---|
| 1 | green | -59.81 | 0.00 | TM |
| 2 | cyan | -59.07 | 0.74 | TM |
| 3 | magenta | -58.60 | 1.20 | ECL2-3 |
| 4 | yellow | -58.49 | 1.32 | VSD4 |
| 5 | sarmon | -58.09 | 1.72 | ECL1-4 |
| 6 | grey | -57.99 | 1.81 | ECL1-4 |
| 7 | skyblue | -57.50 | 2.31 | TM |
| 8 | orange | -57.40 | 2.40 | ECL2-3 |
| 9 | palegreen | -56.66 | 3.14 | VSD4 |
| 10 | deepteel | -56.11 | 3.70 | TM |

**Figure S13.** Superimposed structures of the top 10 models of the original hCa$_v$3.2–hSYN [1–53] and BPP2 complexes with the lowest docking scores.

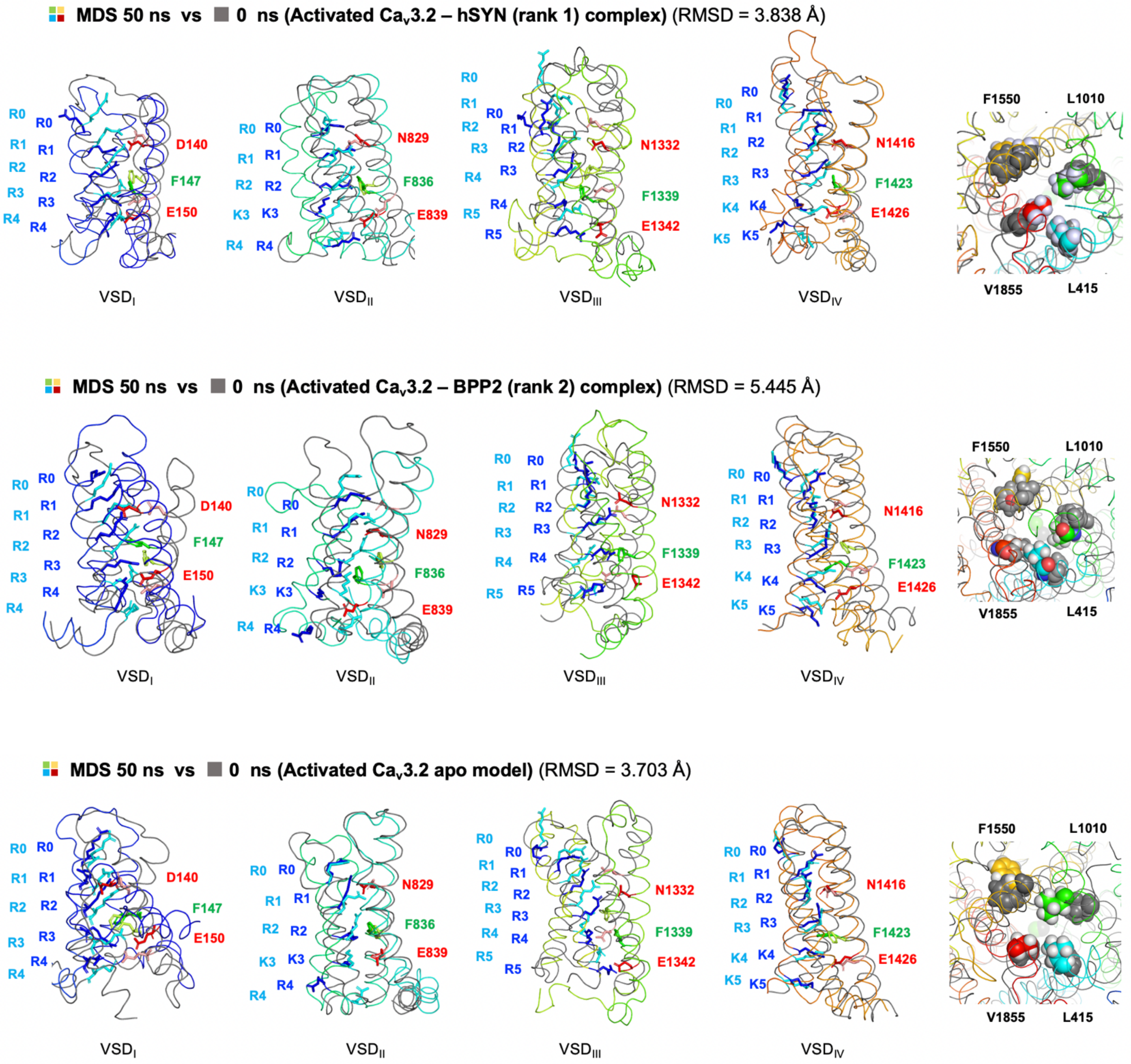


**Figure S14.** Structure comparison of the $VSD_{I\sim IV}$ domains and the bottom internal channel gates of the activated hCa$_v$3.2 – hSYN and BPP2 complexes and its apo hCa$_v$3.2 channel before (grey) and after MDS simulations for 50 ns (multicolor). Characteristic gating positive-charged residues at the S4 helix and their facing residues at the adjacent S2 and S3 helices are highlighted in stick models. The residues on inner channel gate structures (cytosolic view) are shown in sphere models.

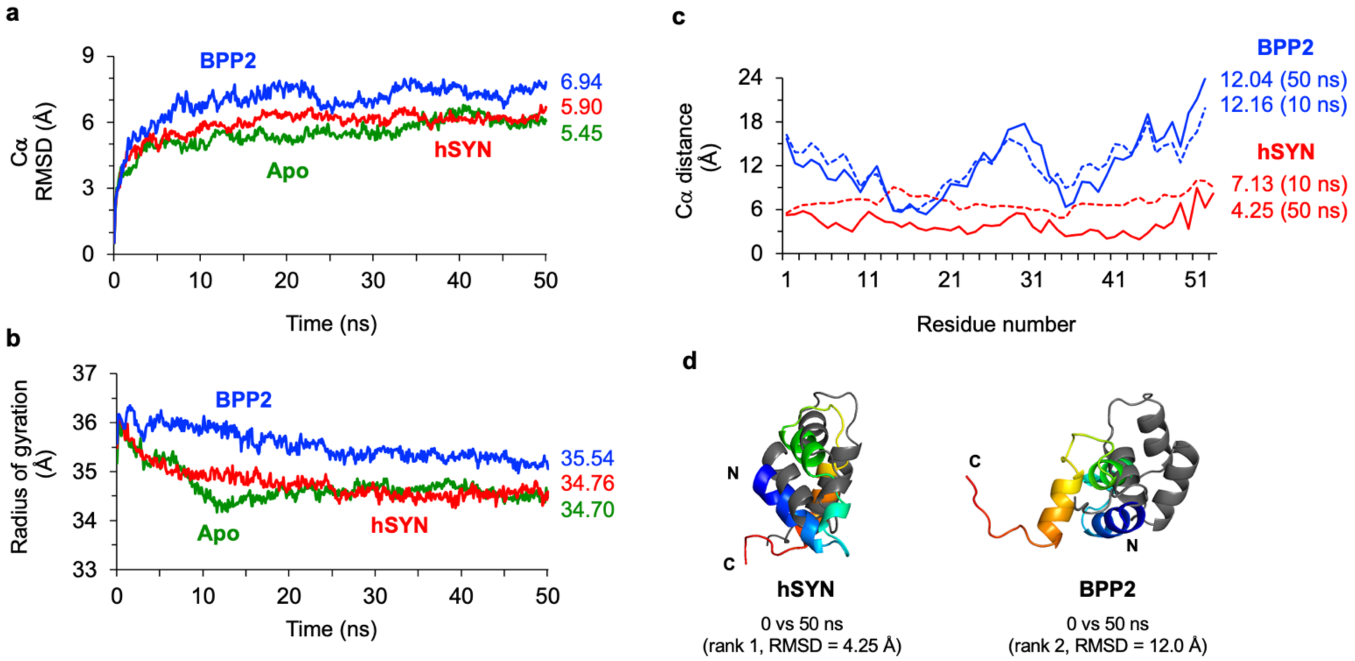


**Figure S15.** MD simulations of the activated hCa$_v$3.2–hSYN [1–53] complex (rank 1), hCa$_v$3.2–BPP2 (rank 2) complex, and the apo hCa$_v$3.2 channel. (a) Conformational stabilities and (b) radius of gyration data for protein compactness simulated at 310 K, pH 7.4 for 50 ns are summarized. Average vales for 50 ns are shown in the right. (c) Conformational stabilities of hSYN (red) and BPP2 (blue) for 10 ns (dashed lines) and 50 ns (solid lines) simulations. (d) Superimposed structures of hSYN and BPP2 on the activated hCa$_v$3.2 model at 0 ns (grey) and 50 ns (multicolor) from the front views. The whole activated hCa$_v$3.2 complexes are shown in Figures 5a and 5b.

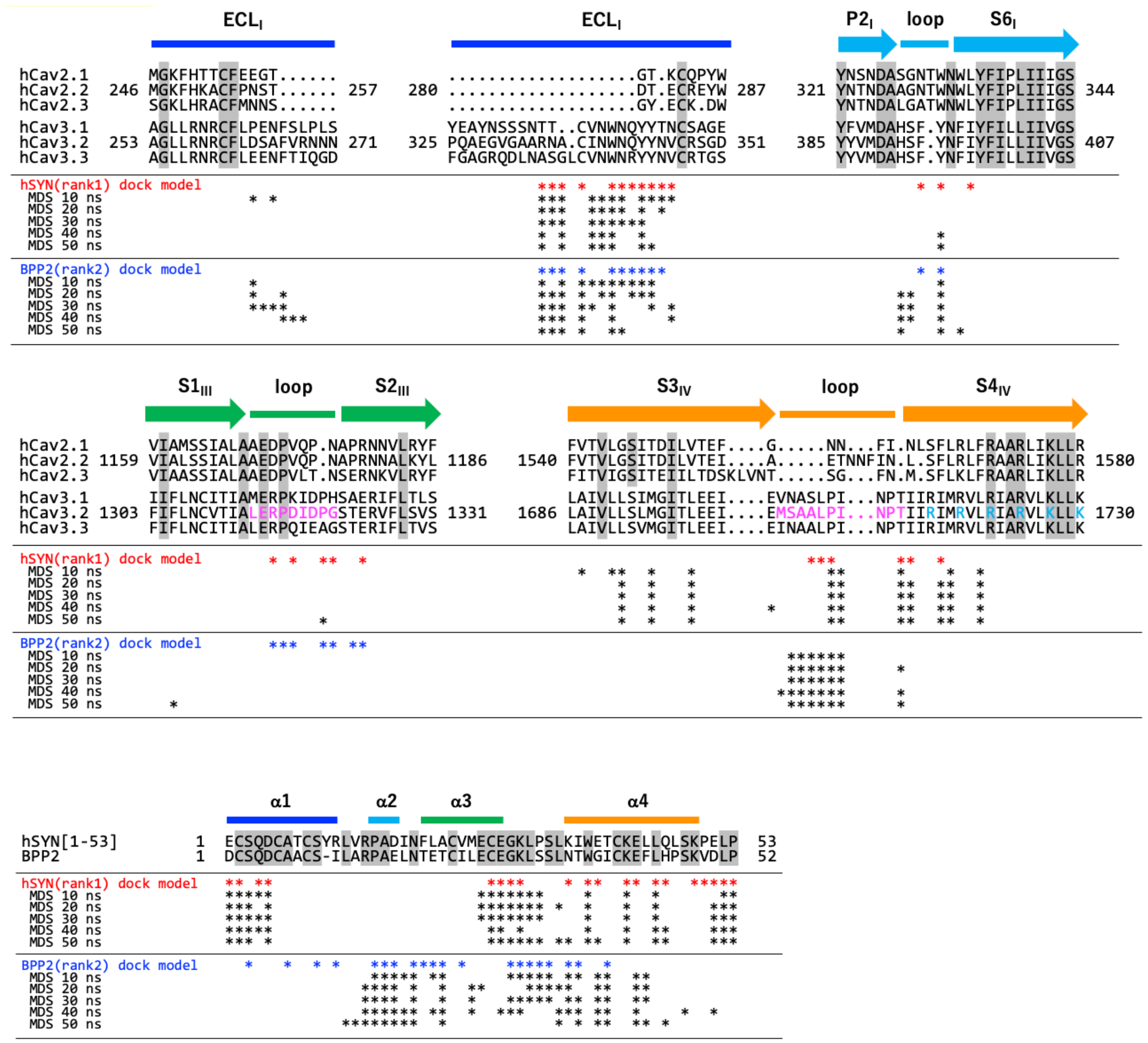


**Figure S16.** PPI analysis. Interacted residues on the activated hCa$_v$3.2 channel and the ligands (hSYN and BPP2) on the complexes are shown in *. The partial amino acid sequences of six channels of hCa$_v$2.1~2.3, 3.1~3.3 are aligned, and identical residues among the six channels are highlighted in grey. The dashes represent gaps. The residues of S1–S2 and S3–S4 loops and the voltage sensor domain residues in hCa$_v$3.2 channel are shown in magenta and cyan, respectively.

## Materials and methods

### General.

An ODS silica gel COSMOSIL75C$_{18}$OPN (Nacalai Tesque) was used for column chromatography. Matrix-assisted laser desorption/ionization with time-of-flight mass spectrometry (MALDI-TOF MS) and a tandem MALDI MS/MS analysis were performed using a Bruker UltrafleXtreme spectrometer equipped with a 355 nm Nd:YAG laser (Smartbeam 1000 or 200 MHz), with α-cyano-4-hydroxycinnamic acid (CHCA) as a matrix.

### Mealworm bioassay

Paralytic activity against mealworms was examined by using synthetic **2** dissolved in saline with mealworms (larva of *Zophobas atratus*, body weight: 0.7–1.0 g), as described previously.[28] Synthetic hSYN was injected into the abdomen of mealworms and observed immediate lower-body paralysis (injection volume: 100 µL per 1.0 g mealworm bodyweight). Phosphate buffered saline (PBS) was used as a control.

### Electrophysiology

Electrophysiological assays were done on human $Ca_v3.2$ channel, which was expressed on human embryonic kidney 293T (HEK293T) cells using Lipophectamine® 3000 transfection reagent. A pcDNA3 plasmid construct containing the complete sequence coding the human α1H-subunit of the $Ca_v3.2$ channel flanked by a green fluorescent protein was kindly provided by Prof. Edward Perez–Reyes (University of Virginia, Charlottesville, Virginia, USA).[S1] Voltage-gated $Ca^{2+}$ currents were recorded by the whole-cell patch-clamp technique, as previously described.[28,S2] This recording was performed in the whole-cell configuration using an Axopatch 200B Amplifier (Axon CNS, Molecular Devices) at a holding potential of –90 mV. Cells were depolarized from the –90 mV holding potential to +80 mV with +10 mV voltage steps of 200 ms. The current amplitude was measured at the peak. The current density was determined with the cell capacitance to establish the density-voltage curves. The currents were filtered at 2 kHz and sampled at 5 kHz using an A/D converter, the Digidata 1440A (Axon CNS, Molecular Devices). The leak was subtracted automatically by a P/4 protocol (pclamp10, Axon CNS, Molecular Devices). For the recording $Ca^{2+}$ currents, the bath solution contained 125 mM *N*-methyl-D-glucamine, 5 mM 4-aminopyridine, 20 mM tetraethylammonium chloride, 2 mM $CaCl_2$, 2 mM $MgCl_2$, and 10 mM D-glucose and was buffered to pH 7.4 with 10 mM HEPES. The patch pipettes were filled with solution containing 130 mM CsCl, 10 mM EGTA, 3 mM Mg–ATP, and 0.4 mM Li–GTP with the pH adjusted to 7.2 by 25 mM HEPES. Statistical analyses were performed using Dunnett's multiple comparison tests. *, $p < 0.05$ versus control.

### Circular dichroism spectrometry and stereostructure prediction of hSYN.

Circular dichroism (CD) spectrum was measured by a JASCO J-1500 CD spectrometer at 25 °C, as described previously.[28] In brief, synthetic hSYN [1–53] (**2**) was dissolved in 50 mM $KHPO_4$, pH 7.0 (final concentration: 20 µM) in a 1 mm pathlength cell. The spectrum consisted of five scans acquired with a scan rate of 20 nm/min and a digital integration time of 2 sec. A spectrum of the buffer recorded under the same conditions was subtracted from that of the sample. The percent helicity was calculated from the observed molar CD data using the K2D3 (190~240 nm) and the BestSel (190~260 nm) web servers.[69,70]

Three-dimension structures of hSYN and BPP2 were constructed by using ColabFold, a stereostructure prediction program based on the amino acid sequences.[64–66] The calculated CD spectrum of **2** was obtained by using the PDBMD2CD server,[68] and its percent helicity was calculated similarly as above. PyMol (Molecular Graphics System, Version 2.0 Schrödinger, LLC) was used for calculation of surface charge and final image processing which suitable for scientific

representation.

**Molecular modeling studies.**

Molecular modeling studies were performed using the Molecular Operating Environment (MOE) 2022.02 program package (Chemical Computing Group, Inc.), similarly as described previously.[S3,S4] To obtain the three-dimensional structures of hCa$_v$3.2 channel, homology modeling was employed using human Na$_v$1.4 (activated state, PDB: 6AGF), American cockroach *Periplaneta americana* Na$_v$1.4 (Na$_v$PaS, inactivated state, PDB: 5X0M), and Campylobacteria *Arcobacter butzleri* Na$_v$ (Na$_v$Ab, resting state, PDB: 6P6W) channels as templates, respectively. The target sequence was aligned with the template using multiple sequence alignment tools, and the models were generated by optimizing the backbone and side-chain conformations, and refined using energy minimization. Since the Na$_v$Ab structure does not contain ECL domain, we complemented the resting models by the replacement with that of hCa$_v$3.2 original structure.

For docking model studies, the phospholipids and $Ca^{2+}$ ions present in the original cryo-EM structure of Ca$_v$3.2 channel were added to the activated, inactivated, and resting models obtained as described above. Conformational searches were performed using the Amber14:EHT force-field with GB/VI Generalized Born implicit solvent electrostatics ($D_{in}$ = 1, $D_{out}$ = 80) and with LowModeMD, in which the S1–S2 and S3–S4 loops on the Ca$_v$3.2 channels were settled as the interaction sites with peptide ligands. Refinements were performed using a rigid-body model, and the lowest conformation models in energy for hSYN (or the second lowest model for BPP2) were used for further MD simulation studies.

**Molecular dynamics simulation.** Molecular dynamics (MD) simulations were performed for the activated hCa$_v$3.2 – hSYN and – BPP2 complexes using YASARA (21.12.19) software to evaluate the conformational stability of the receptors and ligands.[S5] The complexes obtained by PPI-Dock simulations as described above were inserted into a cubic box of water molecules, with a density of 0.997 g/mL and a temperature of 310 K. We used 0.9% NaCl (physiological solution) and a default physiological pH at 7.4. The simulation was performed at a normal speed (2 × 1.25 fs timestep). After the steepest descent and simulated annealing minimizations, a simulation was run for 50 ns using the AMBER14 Force Field. The water molecules were described by the TIP3P model. Periodic boundary conditions were applied. All protein structural data were represented in cartoon and surface model, while ligand was showed as sphere or stick model. The interacted residues of hCa$_v$3.2 channel with hSYN and BPP2 were visualized with the Pymol software.

**Automated solid phase peptide synthesis.**

Peptides were synthesized on a CSBio 136X synthesizer using a standard Fmoc SPPS chemistry, as described previously.[28] The following Fmoc amino acids with the protected side-chains were used: Fmoc-Ala-OH, Fmoc-Arg(Pbf)OH, Fmoc-Asn(Trt)-OH, Fmoc-Asp(O$^t$Bu)-OH, Fmoc-Cys(Trt)-OH, Fmoc-Cys(Acm)-OH, Fmoc-Cys($^t$Bu)-OH, Fmoc-Gln(Trt)-OH, FmocGlu(O$^t$Bu)-OH, Fmoc-Gly-OH, Fmoc-His(1-Trt)-OH, Fmoc-Ile-OH, Fmoc-Leu-OH, Fmoc-Lys(Boc)OH, Fmoc-Phe-OH, Fmoc-Pro-OH, Fmoc-Ser($^t$Bu)-OH, Fmoc-Thr($^t$Bu)-OH, Fmoc-Trp(Boc)-OH, Fmoc-Tyr($^t$Bu)-OH, and Fmoc-Val-OH. SPPS was performed with a 2-chlorotrityl chloride resin or an H-Ala-hydrazide preloaded 2-chlorotrityl resin. The first residue was manually loaded onto the resin and subsequent Fmoc-SPPS followed the standard protocols. Fmoc-deprotections were performed with 20% (v/v) piperidine in DMF (8 min × 2). Couplings were performed with Fmoc-amino acid (4.0 equiv relative to resin substitution), HCTU (3.8 equiv) and NMM (8.0 equiv) in DMF for 60 min. If required, the coupling step was repeated (double coupling) and LiCl washes (0.8 M LiCl in DMF) were performed before the next step. After coupling, unreacted free amine was capped by treatment with 20% (v/v) acetic anhydride and 10% (v/v) NMM in DMF for 10 min. For the coupling with the residues prone to epimerization such as cysteine, HOBt (4.0 equiv) and DIC (4.0 equiv) were in DMF

were used, and the reaction time was extended to 2 h. After the peptide elongation, the resin was washed several times with DMF followed by $CH_2Cl_2$, dried and treated with a TFA cleavage cocktail (TFA/phenol/$H_2O$/TIPS = 88/5/5/2, vol/wt/vol/vol, 30 mL/g resin) for 3 h at room temperature. After filtration, the filtrate was concentrated under reduced pressure. The residue was triturated with cooled $Et_2O$, centrifuged and the supernatant was removed by decantation. This trituration/decantation step was repeated three times. The obtained crude peptide was dissolved in 0.1 % aq. TFA, lyophilized, and purified with RP-HPLC.

**Preparation and disulfide bond connectivity analysis of hSYN [1–53] 2 (Type II).**

**N-terminal [1–23] hydrazide segment**. Prepared from the H-Ala-$NHNH_2$ preloaded 2-chloro trityl resin (50 mg, 70 μmol, loading: 0.35 mmol/g) by automated Fmoc SPPS method. The crude peptide was purified by RP-HPLC using a Develosil ODS HG-5 column (5 μm, ϕ 20 mm × 250 mm) at 27 °C with a linear gradient of 20–50% aq. MeCN / 0.1% TFA for 113 min, at a flow rate of 5 mL/min to N-terminal segment (25 mg, 14%) as a white powder.

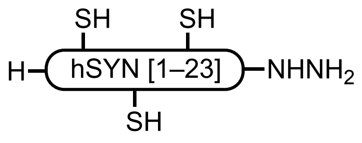


N-terminal segment

N-terminal [1–23] hydrazide segment: $t_R$ 17.8 min [Develosil ODS-HG-5 (ϕ 4.6 × 250 mm), temp. 27 °C, 20–60% linear gradient for 40 min, flow 1.0 mL/min, UV 215 nm (A; 0.1% aq. TFA, B; 0.08% TFA/MeCN)]. MS (MALDI-TOF) *m/z* 2589.3 (calcd for $C_{108}H_{175}N_{33}O_{35}S_3$ $[M+H]^+$, Δ +0.1 mu).

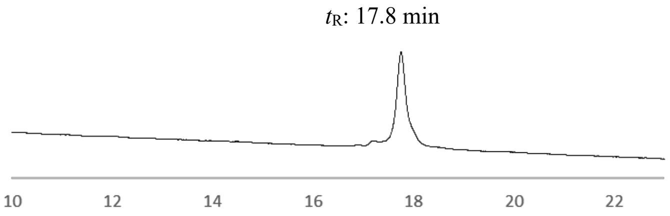


**C-terminal [24–53] cysteine segment**. Prepared from Fmoc-Pro-OH preloaded 2-chloro trityl resin (36 mg, 50 μmol, loading: 0.29 mmol/g) by automated Fmoc SPPS method. The crude peptide was purified by RP-HPLC using a Develosil ODS HG-5 column (5 μm, ϕ 20 mm × 250 mm) at 27 °C with a linear gradient of 30–60% aq. MeCN / 0.1% TFA for 113 min, at a flow rate of 5 mL/min to C-terminal segment (10 mg, 6%) as a white powder.

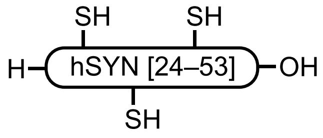


C-terminal segment

C-terminal [24–53] cysteine segment: $t_R$ 18.2 min [Develosil ODS-HG-5 (ϕ 4.6 × 250 mm), temp. 27 °C, 30–60% linear gradient for 20 min, flow 1.0 mL/min, UV 215 nm (A; 0.1% aq. TFA, B; 0.08% TFA/MeCN)]. MS (MALDI-TOF) *m/z* 3444.8 (calcd for $C_{153}H_{256}N_{36}O_{45}S_4$ $[M+H]^+$, Δ +0.0 mu).

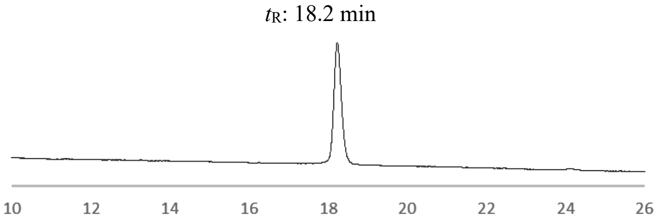

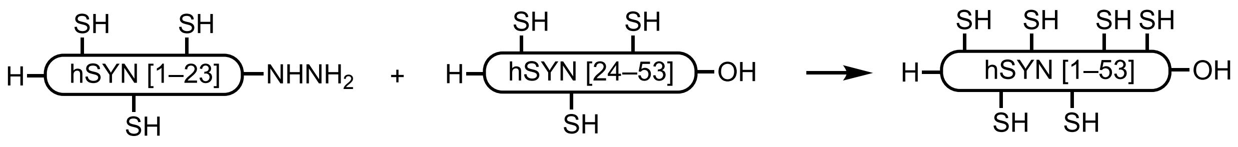


N-terminal segment C-terminal segment **1**

**Linear peptide 1**. N-terminal [1–23] hydrazide segment (4.5 mg, 1.7 µmol) was dissolved in 6 M Gdm-Cl (290 µL, pH 3.0), then 10% aq. acetylacetone (17.8 µL, 17.4 µmol) and MPAA (5.4 mg, 35 µmol) were added. After stirring at room temperature for 1 h, C-terminal [24–53] cysteine segment (7.2 mg, 2.1 µmol), and TCEP·HCl (0.32 mg, 1.1 µmol) dissolved in 6 M Gdm-Cl / 0.2 M $NaH_2PO_4$ (290 µL, pH 8.5) were added, and adjusted to pH 7.0 with 6 M aq. NaOH. After standing at room temperature for 22 h, the reaction mixture was purified by an ODS open column (Cosmosil® $75C_{18}$-OPN, 0.2 g, 0 to 50% aq. MeCN / 0.1% TFA) and RP-HPLC using a Develosil® ODS HG-5 column (5 µm, ϕ 20 mm × 250 mm) at 27 °C with a linear gradient of 30–60% aq. MeCN / 0.1% TFA for 113 min, at a flow rate of 5 mL/min to give linear peptide **1** (4.9 mg, 48%) as a white powder.

**1**: $t_R$ 22.3 min [Develosil® ODS HG-5 (5 µm, ϕ 4.6 mm × 250 mm), temp. 27 ºC, 30–60% linear gradient for 30 min, flow rate 1.0 mL/min, UV 215 nm (A; 0.1% aq. TFA, B; 0.08% TFA/MeCN)]. MS (MALDI-TOF) *m/z* 3001.0 (calculated for $C_{261}H_{420}N_{67}O_{80}S_7$ $[M+2H]^{2+}$ , Δ +0.0 mu)

$t_R$: 22.3 min

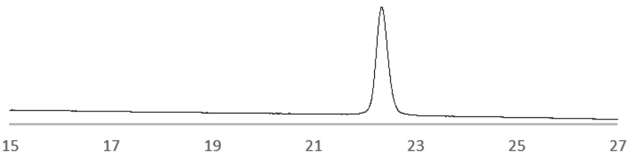


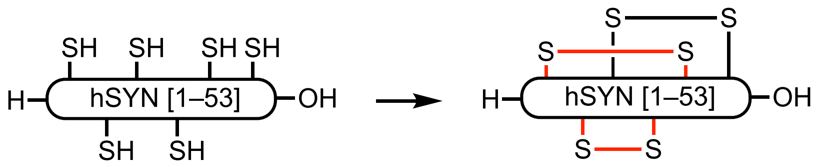


**1** **2** (Type II)

**Type II hSYN [1–53] (2)**. Folding of the linear BPP2 was conducted using a cysteine/cystine redox system (Figures S1 A,B, entry 3). Linear peptide **1** (1.0 mg, 0.17 µmol) was dissolved in the refolding buffer (5 mM cysteine / 0.5 mM cystine in 20% EtOH / 0.1 M aq. $NH_4OAc$, pH 8.5, 2.7 mL) under a nitrogen atmosphere. After stirring at 37 ºC for 72 h, the reaction mixture was lyophilized, and purified by RP-HPLC using a Develosil® ODS HG-5 column (5 µm, ϕ 20 mm × 250 mm) at 25 ºC with a linear gradient of 30–60% aq. MeCN / 0.1% TFA for 56.7 min, at a flow rate of 5.0 mL/min to afford Type II hSYN [1–53] (**2**) (0.66 mg, 65%) as a white powder. Refolding of the misfolded 3SS products (Figure S1C) and purified **2** (Figure S1D) were conducted using the same cysteine/cystine redox condition as mentioned above.

**2**: $t_R$ 12.2 min [Develosil® ODS HG-5 (5 µm, ϕ 4.6 mm × 250 mm), temp. 25 ºC, 30–60% linear gradient for 30 min, flow rate 1.0 mL/min, UV 215 nm (A; 0.1% aq. TFA, B; 0.08% TFA/MeCN), see Figure S1D]; $t_R$ 18.8 min [Agilent ZORBAX SB-C18 (3.5 µm, ϕ 0.5 mm × 150 mm), temp. 40 ºC, 25% for 10 min and 25–45% linear gradient for 20 min, flow rate 16 µL/min, UV215 nm (A; 0.1% aq. TFA, B; 0.08% TFA/MeCN), see Figure S7]; MS (MALDI-TOF) *m/z* 2998.0 (calculated for $C_{261}H_{420}N_{67}O_{80}S_7$ $[M+2H]^{2+}$, Δ –0.4 mu).

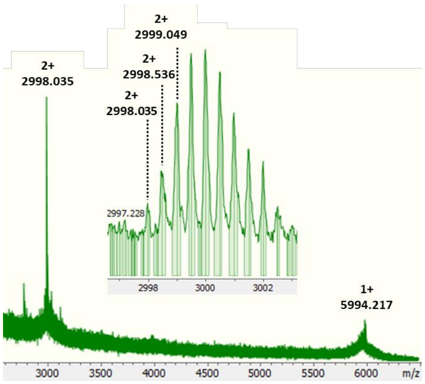


MALDI-TOF MS

**Intact enzymatic degradation of 2 and 10.** Synthetic Type-II hSYN [1–53] (**2**) (50 µg, 8.4 nmol) was treated with trypsin or Glu-C (6.7 ng/µL) in 50 mM $NH_4HCO_3$ aq. (30 µL) at 37 °C for 28 h. The resulting mixture was quenched with 10% TFA aq. (3 µL), desalted with Zip-Tip® $C_{18}$, and analyzed by MALDI-MS (Figures S2 and S3). The Glu-C treated **2** and **10** were also analyzed by OrbiTrap Exploris 240 LC-MS (Thermo) (Figure S7c).

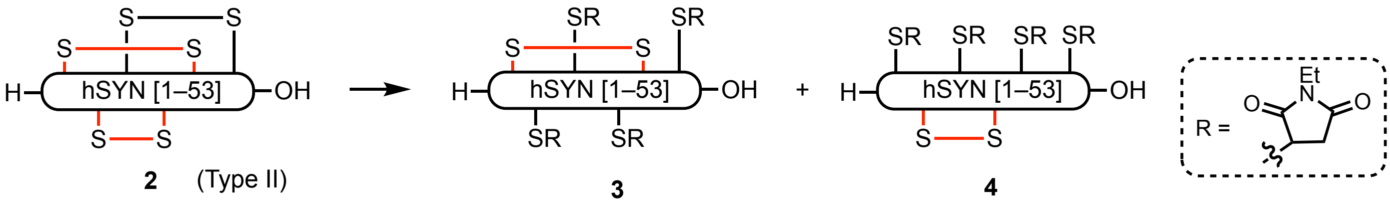


**Partial reduction and NEM-alkylation.** Synthetic Type-II hSYN [1–53] (**2**) (25 µg, 4.2 nmol) was denatured with 6 M Gdm-Cl in 0.1 M citrate (4.4 µL, pH 3.0) at 65 °C for 20 min. Subsequently, 10 mM TCEP·HCl in the above buffer (1.3 µL, pH 3.0) was added. After incubating at 65 °C for 15 min, 0.2 M NEM in 0.1 M citrate (5.3 µL) was added. The resulting mixture was incubated at 27 °C for 20 min and directly purified by RP-HPLC using a Develosil ODS-HG-5 column (5 µm, Φ 4.6 mm × 250 mm) at 27 °C with a linear gradient of 30–60% for 60 min, flow rate 1.0 mL/min, UV 215 nm (A; 0.1% aq. TFA, B; 0.08% TFA/MeCN) to give 2SS-a/b, 1SS-a (**3**), 1SS-b (**4**), 0SS (**1**), and unreacted 3SS (**2**), which were concentrated and lyophilized for further degradation reactions.

**3**: $t_R$ 29.6 min [same as above]. MS (MALDI-TOF): *m/z* 3250.3 (calculated for $C_{285}H_{452}N_{71}O_{88}S_7$ $[M+2H]^{2+}$, Δ –0.3 mu)

**4**: $t_R$ 37.3 min [same as above]. MS (MALDI-TOF): *m/z* 3250.5 (calculated for $C_{285}H_{452}N_{71}O_{88}S_7$ $[M+2H]^{2+}$, Δ –0.1 mu)

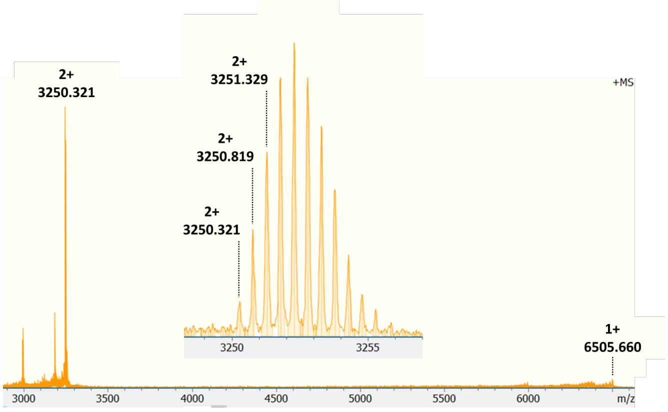


MALDI-TOF MS of **3**

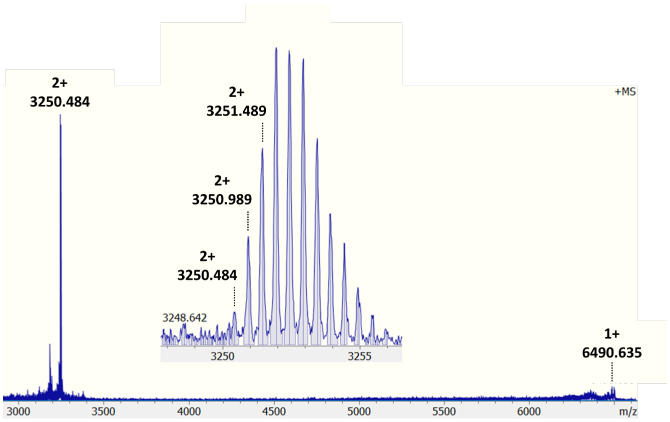


MALDI-TOF MS of **4**

**Full reduction, Cam-alkylation and tryptic digestions of 1SS analogues.** To the solutions of **3** and **4** in 52.6 mM aq. $NH_4HCO_3$ (10 μL) was added 45 mM DTT (2 μL). After incubation at 50 °C for 15 min, 0.1 M iodoacetamine (IAM) in 52.6 mM aq. $NH_4HCO_3$ (1.0 μL) was added. After incubation at 27 °C for 15 min, the resulting mixture was diluted with 52.6 mM aq. $NH_4HCO_3$ (16 μL) and treated with trypsin (50 ng, 1 μL in DDW). After incubation at 37 °C for 24 h, the resulting peptide mixture were analyzed by MALDI-TOF MS and MS/MS after desalting with a ZipTipC$_{18}$ tip column. (The detailed data are shown in Figures S4–S6)

**Preparation of (Type I) hSYN [1–53] (10).**

**N-terminal segment [1–23] 5**. Prepared from the H-Ala-$NHNH_2$ preloaded 2-chloro trityl resin (50 mg, 70 μmol, loading: 0.35 mmol/g) as mentioned above. The crude peptide was purified by RP-HPLC using a Develosil ODS HG-5 column (5 μm, ϕ 20 mm × 250 mm) at 27 °C with a linear gradient of 30–60% aq. MeCN / 0.1% TFA for 113 min, at a flow rate of 5 mL/min to N-terminal segment [1-23] **5** (9.5 mg, 3%) as a white powder.

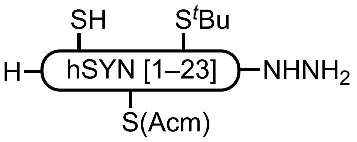


**5**

**5**: $t_R$ 12.1 min [Develosil ODS-HG-5 (ϕ 4.6 × 250 mm), temp. 27 °C, 25–60% gradient for 35 min, flow 1.0 mL/min, UV 215 nm (A; 0.1% aq. TFA, B; 0.08% TFA/MeCN)]. MS (MALDI-TOF) *m/z* 2716.4 (calcd for $C_{115}H_{188}N_{34}O_{36}S_3$ $[M+H]^+$, Δ +0.1 mu).

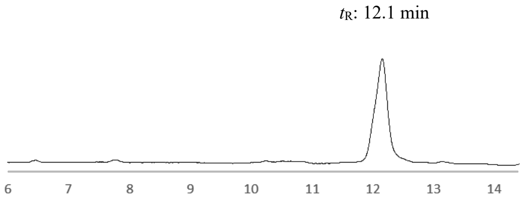


**C-terminal segment [24–53] 6**. Prepared from Fmoc-Pro-OH preloaded 2-chloro trityl resin (36 mg, 50 μmol, loading: 0.29 mmol/g) as mentioned above. The crude peptide was purified by RP-HPLC using a Develosil ODS HG-5 column (5 μm, ϕ 20 mm × 250 mm) at 27 °C with a linear gradient of 40–60% aq. MeCN / 0.1% TFA for 60 min, at a flow rate of 5 mL/min to C-terminal segment [24–53] **6** (8.9 mg, 5%) as a white powder.

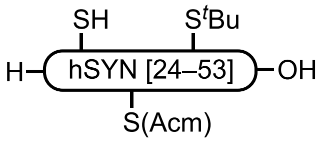


**6**

**6**: $t_R$ 17.7 min [Develosil ODS-HG-5 (ϕ 4.6 × 250 mm), temp. 27 °C, 30–50% gradient for 20 min, flow 1.0 mL/min, UV 215 nm (A; 0.1% aq. TFA, B; 0.08% TFA/MeCN)]. MS (MALDI-TOF) *m/z* 3571.9 (calcd for $C_{160}H_{269}N_{37}O_{46}S_4$ $[M+H]^+$, Δ +0.0 mu).

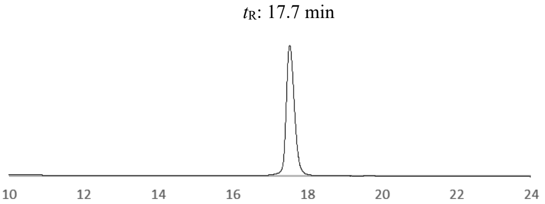

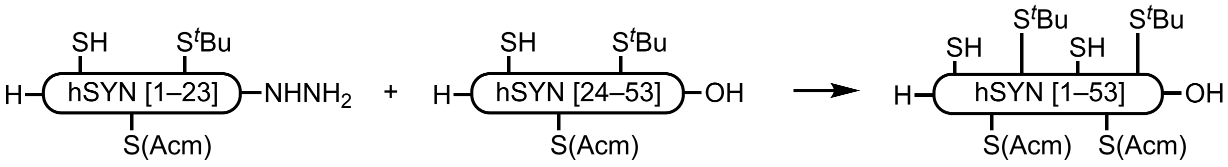


**5** **6** **7**

**Linear peptide 7**. Prepared from N-terminal segment [1–23] **5** (1.1 mg, 320 nmol) and C-terminal segment [24–53] **6** (1.6 mg, 380 nmol) using the same procedure as that for **1**. The reaction mixture was purified by an ODS open column (Cosmosil® $75C_{18}$-OPN, 0.1 g, 0 to 50% aq. MeCN / 0.1% TFA) and RP-HPLC using a Develosil® ODS HG-5 column (5 µm, ϕ 20 mm × 250 mm) at 27 °C with a linear gradient of 30–60% aq. MeCN / 0.1% TFA for 113 min, at a flow rate of 5 mL/min to give linear peptide **1** (1.0 mg, 40%) as a white powder.

**7**: $t_R$ 21.7 min [Develosil ® ODS HG-5 (5 µm, ϕ 4.6 mm × 250 mm), temp. 27 ºC, 20–60% linear gradient for 30 min, flow 1.0 mL/min, UV 215 nm (A; 0.1% aq. TFA, B; 0.08% TFA/MeCN). MS (MALDI-TOF) *m/z* 3128.5 (calculated for $C_{275}H_{451}N_{69}O_{82}S_7$ $[M+2H]^{2+}$, Δ –0.1 mu)

$t_R$: 21.7 min

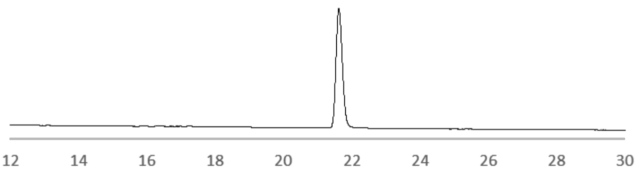


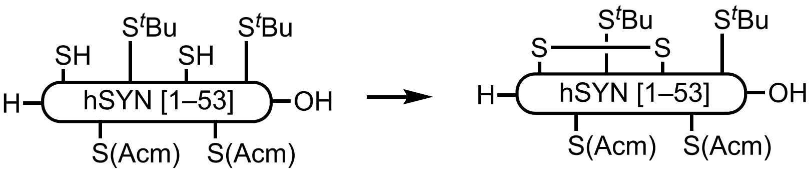


**7** **8**

**1SS intermediate 8.** A solution of linear peptide 7 (1.8 mg, 290 nmol) was dissolved in H2O/MeCN/DMSO (15:10:1, 5.6 mL) under a nitrogen atmosphere. After stirring at room temperature for 36 h, the resulting mixture was lyophilized and purified by RP-HPLC using a Develosil® ODS HG-5 column (5 µm, ϕ 20 mm × 250 mm) at 27 °C with a linear gradient of 40–60% aq. MeCN / 0.1% TFA for 75.6 min, at a flow rate of 5 mL/min to give 1SS intermediate **8** (1.1 mg, 40%) as a white powder.

**8**: $t_R$ 13.5 min [Develosil ODS-HG-5 (ϕ 4.6 mm × 250 mm), temp. 27 °C, 40–60% gradient for 20 min, flow 1.0 mL/min, UV 215 nm (A; 0.1% aq. TFA, B; 0.08% TFA / MeCN)]. MS (MALDI-TOF) *m/z* 3127.7 (calcd for $C_{275}H_{452}N_{69}O_{82}S_7$ $[M+2H]^{2+}$, Δ –0.1 mu).

$t_R$: 13.5 min

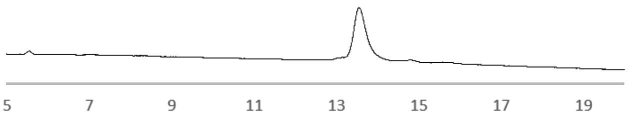

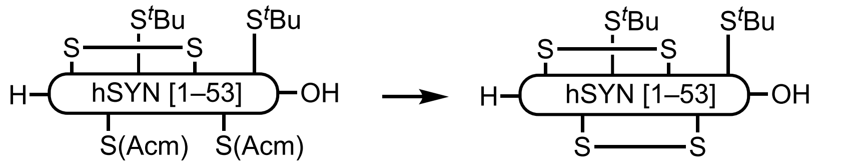


**8** **9**

**2SS intermediate 9.** To the solution of **8** (0.60 mg, 90 nmol) in MeOH/AcOH (1:1, 0.30 mL) was added iodine (113 µg, 0.90 mmol) under a nitrogen atmosphere. After stirring for 30 min at room temperature, 1 M sodium ascorbate (30 µL) was added. After stirring for 1 h, the resulting mixture was purified by RP-HPLC using a Develosil® ODS HG-5 column (5 µm, ϕ 10 mm × 250 mm) at 27 °C with a linear gradient of 40–60% aq. MeCN / 0.1% TFA for 47.8 min, at a flow rate of 2 mL/min to give 2SS intermediate **9** (0.40 mg, 67%) as a white powder.

**9**: $t_R$ 31.9 min [Develosil XG-C18M (ϕ 2.0 mm × 150 mm), temp. 27 °C, 40–60% gradient for 40 min, flow 0.2 mL/min, UV 215 nm (A; 0.1% aq. TFA, B; 0.08% TFA / MeCN)]. MS (MALDI-TOF) *m/z* 3070.6 (calcd for $C_{269}H_{438}N_{67}O_{80}S_7$ $[M+2H]^{2+}$, Δ +0.1 mu)

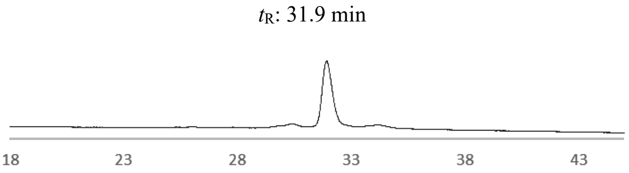


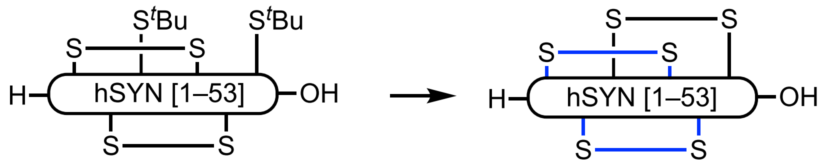


**9** **10** (Type I)

**Type I hSYN [1–53] (10).** A solution of **9** (0.20 mg, 32 nmol) in TFA / DMSO (37:1, 195 µL) was stirred for 1 h at room temperature under a nitrogen atmosphere. The resulting mixture was diluted with water (1 mL), lyophilized, and purified by RP-HPLC using a Develosil® ODS HG-5 column (5 µm, ϕ 4.6 mm × 250 mm) at 27 °C with a linear gradient of 30–60% aq. MeCN / 0.1% TFA for 30 min, at a flow rate of 1 mL/min to give Type I hSYN [1–53] **10** (20 µg, 10%) as a white powder.

**10**: $t_R$ 17.6 min [Agilent ZORBAX SB-C18 (ϕ 0.5 mm × 150 mm, 3.5 µm), temp. 40 °C, 25% isocratic for 10 min and 25–45% gradient for 20 min, flow 16 µL/min, UV 214 nm (A; 0.1% aq. TFA, B; 0.08% TFA / MeCN), see Figure S7]; MS (MALDI-TOF) *m/z* 2998.1 (calcd for $C_{261}H_{420}N_{67}O_{80}S_7$ $[M+2H]^{2+}$, Δ –0.3 mu).

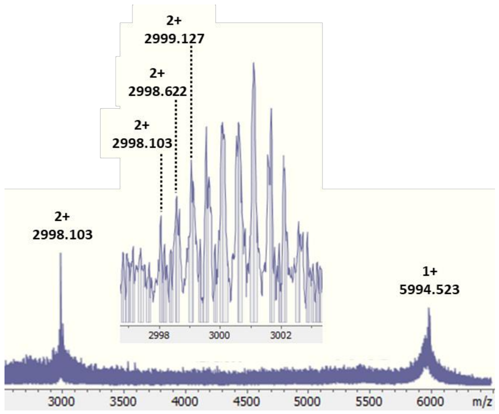


MALDI-TOF MS

**Supporting references**


S1. T. Mannic, M. Mouffok, M. Python, T. Yoshida, A. D. Maturana, N. Vuilleumier, M. F. Rossier, *Endocrinology* **2013**, *154*, 1271–1281.

S2. J. Ito, T. Minemura, S. Wälchli, T. Niimi, Y. Fujihara, S. Kuroda, K. Takimoto, A. D. Maturana, *Int. J. Mol. Sci.* **2021**, *22*, 3561.

S3. M. Itakura, D. H. Utomo, M. Kita, *Chem. Commun.* **2024**, *60*, 4910–4913.

S4. Y. Sun, A. Dakiiwa, M. Zhang, T. Shibata, M. Kita, *Chem. Eur. J.* **2024**, *30*, e202402049.

S5. H. Land, M. S. Humble, *Methods Mol. Biol.* **2018**, *1685*, 43–67.
